# Rare Variant Burden Analysis within Enhancers Identifies *CAV1* as an ALS Risk Gene

**DOI:** 10.1016/j.celrep.2020.108456

**Published:** 2020-12-01

**Authors:** Johnathan Cooper-Knock, Sai Zhang, Kevin P. Kenna, Tobias Moll, John P. Franklin, Samantha Allen, Helia Ghahremani Nezhad, Alfredo Iacoangeli, Nancy Y. Yacovzada, Chen Eitan, Eran Hornstein, Eran Ehilak, Petra Celadova, Daniel Bose, Sali Farhan, Simon Fishilevich, Doron Lancet, Karen E. Morrison, Christopher E. Shaw, Ammar Al-Chalabi, Project MinE ALS Sequencing Consortium, Ian Blair, Naomi Wray, Matthew Kiernan, Miguel Mitne Neto, Adriano Chio, Ruben Cauchi, Wim Robberecht, Philip van Damme, Phillippe Corcia, Phillipe Couratier, Orla Hardiman, Russel McLaughlin, Marc Gotkine, Vivan Drory, Nicola Ticozzi, Vincenzo Silani, Jan Veldink, Leonard van den Berg, Mamede de Carvalho, Jesus Mora Pardina, Monica Povedano, Peter Andersen, Markus Wber, Nazli Başak, Ammar Al-Chalabi, Christopher Shaw, Pamela Shaw, Karen Morrison, John Landers, Jonathan Glass, Jan H. Veldink, Janine Kirby, Michael P. Snyder, Pamela J. Shaw

**Affiliations:** 1 Sheffield Institute for Translational Neuroscience (SITraN), University of Sheffield, Sheffield, UK; 2 Stanford Center for Genomics and Personalized Medicine, Department of Genetics, Stanford University School of Medicine, Stanford, CA 94305, USA; 3 Department of Neurology, Brain Center Rudolf Magnus, University Medical Center Utrecht, Utrecht, the Netherlands; 4 Department of Basic and Clinical Neuroscience, Institute of Psychiatry, Psychology and Neuroscience, King’s College London, London, UK; 5 Department of Molecular Genetics, Weizmann Institute of Science, Rehovot, Israel; 6 Department of Biology, Lund University, Lund, Sweden; 7 Sheffield Institute for Nucleic Acids, University of Sheffield, Sheffield, UK; 8 Analytic and Translational Genetics Unit, Department of Medicine, Massachusetts General Hospital and Harvard Medical School, Boston, MA, USA; 9 Faculty of Medicine, University of Southampton, Southampton, UK

**Keywords:** amyotrophic lateral sclerosis, whole-genome sequencing, CAV1, CAV2, non-coding DNA, gene enhancers, membrane lipid rafts

## Abstract

Amyotrophic lateral sclerosis (ALS) is an incurable neurodegenerative disease. CAV1 and CAV2 organize membrane lipid rafts (MLRs) important for cell signaling and neuronal survival, and overexpression of CAV1 ameliorates ALS phenotypes *in vivo*. Genome-wide association studies localize a large proportion of ALS risk variants within the non-coding genome, but further characterization has been limited by lack of appropriate tools. By designing and applying a pipeline to identify pathogenic genetic variation within enhancer elements responsible for regulating gene expression, we identify disease-associated variation within *CAV1/CAV2* enhancers, which replicate in an independent cohort. Discovered enhancer mutations reduce *CAV1/CAV2* expression and disrupt MLRs in patient-derived cells, and CRISPR-Cas9 perturbation proximate to a patient mutation is sufficient to reduce *CAV1/CAV2* expression in neurons. Additional enrichment of ALS-associated mutations within *CAV1* exons positions *CAV1* as an ALS risk gene. We propose *CAV1/CAV2* overexpression as a personalized medicine target for ALS.

## Highlights

• Identification of ALS-associated genetic variation within gene enhancers
• ALS-associated risk variants identified within enhancers and exons linked to *CAV1*
• *CAV1/CAV2* enhancer mutations reduce gene expression and disrupt membrane lipid rafts
• CRISPR-Cas9 perturbation of enhancer reduces *CAV1/CAV2* expression in neurons

## Graphical Abstract

**Figure:**
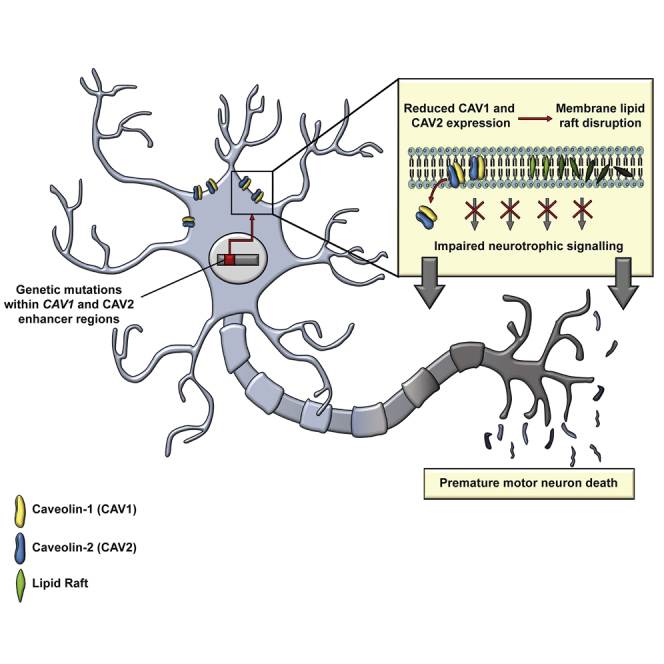

## Teaser

Cooper-Knock et al. identify amyotrophic lateral sclerosis (ALS) risk variants within non-coding regulatory DNA linked to a known ALS gene, *TBK1*, but also *CAV1* and *CAV2*. Disease-associated variants reduce *CAV1/CAV2* expression and disrupt membrane lipid rafts with consequences for neurotrophic signaling. *CAV1* coding sequence also contains ALS-associated mutations.

## Introduction

Amyotrophic lateral sclerosis (ALS) is a universally fatal and relatively common neurodegenerative disease. Progress has been made in identification of highly penetrant coding-sequence mutations responsible for monogenic ALS, but the majority of sporadic ALS patients have no identified genetic risk factor despite heritability estimates as high as 52% (Ryan et al., 2019; Trabjerg et al., 2020). Importantly, discovery of genetic risk factors often leads to therapeutic targets.

ALS is defined by motor neuron death within the CNS; in motor neurons, caveolin 1 (CAV1) and caveolin 2 (CAV2) are expressed together in a hetero-oligomeric complex (de Almeida, 2017) within membrane lipid rafts (MLRs) on the cell surface and have a key role in organization of intercellular signaling (Sawada et al., 2019; Schmick and Bastiaens, 2014). CAV1 activity promotes neurotrophic signaling, leading to enhanced neuronal survival (Head et al., 2011; Mandyam et al., 2017). In contrast, loss of CAV1 accelerates neurodegeneration (Head et al., 2010, 2011). Abnormal neurotrophic signaling is well documented in ALS (Mutoh et al., 2000; Turner et al., 2004), and in particular, deficient neurotrophic signaling is associated with an increased vulnerability to neuronal injury (Bemelmans et al., 2006; Ghavami et al., 2014; Kowiański et al., 2018; Tooze and Schiavo, 2008). There are ongoing efforts to rebalance neurotrophic signaling in ALS patients (Berry et al., 2019); interestingly, neuronal overexpression of CAV1 improves survival and reduces motor neuron death in a mouse model of ALS (Sawada et al., 2019) and is being developed as a therapy for ALS (US patent no. 8969077B2) (Head et al., 2012).

Genome-wide association studies suggest a significant proportion of missing heritability for ALS is distributed throughout non-coding chromosomal regions (van Rheenen et al., 2016). Indeed, a large proportion of human DNA under evolutionary constraint (Ward and Kellis, 2012) is non-coding, and mutations in non-coding DNA affect biological fitness (Graur, 2017), suggesting an important role in all aspects of cellular function. To date, genetic discoveries within the non-coding genome have been limited by a shortage of appropriate methodology.

The non-coding genome contains regions that regulate expression of coding genes; these regions include enhancers, which are *cis*-acting DNA sequences that modulate expression of target genes primarily through binding of transcription factors (TFs) (Koch et al., 2011). Physical interaction between an enhancer and the promoter of the target gene is mediated by DNA looping (Pennacchio et al., 2013). We have designed a pipeline for identification of disease-associated genetic variation within enhancers; variants are aggregated according to function, filtered based on evolutionary conservation (Hujoel et al., 2019), and collapsed into a single burden test (Cirulli and Goldstein, 2010).

We hypothesized that genetic variation within enhancers linked to expression of *CAV1* and *CAV2* would be associated with risk of ALS. Application of our pipeline confirmed our hypothesis and places this pathway upstream in the development of neurodegeneration.

## Results

### Association of Regulatory Enhancer Elements with Coding Genes

Aggregation of genetic material with a common biological function improves power to detect genetic association via burden testing (Cirulli and Goldstein, 2010). We have aggregated sets of enhancers that regulate a common coding gene. As previously described (Fishilevich et al., 2017), we identified high-quality manually curated links between enhancers and coding genes based on agreement between correlated expression between genes, enhancer-RNAs (eRNAs), and TFs; expression quantitative trait loci (eQTL) within enhancers; capture Hi-C; and gene-enhancer genomic distances. Gene-enhancer relationships may be cell type specific (Heinz et al., 2015), whereas our method is cell and tissue agnostic. The disadvantage of this is that highly cell-specific relationships may be missed; however, by including data from multiple cell types, our method benefits from an increase in the quantity of high-quality training data. Moreover, many previously identified ALS-associated mutations are widely expressed (Cooper-Knock et al., 2013). Enhancers linked to *CAV1* and *CAV2* are detailed in Table S1.

### A Pipeline for Testing for Disease-Associated Genetic Variation within Enhancers

**Figure 1:**
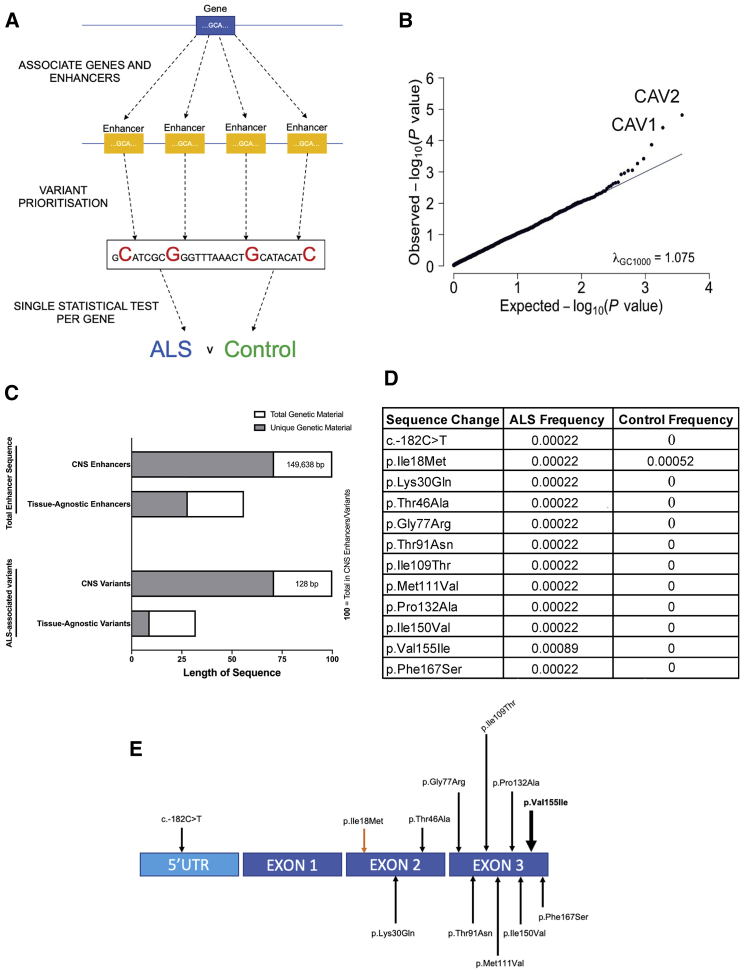
Significant Enrichment of ALS Genetic Risk within Enhancers and Coding Regions Linked to *CAV1* and *CAV2* (A) Pipeline for variant filtering and burden testing; enhancers are first associated with genes based on epigenetic and transcriptome data (Fishilevich et al., 2017); enhancer variants are prioritized for further analysis if they are rare (MAF < 0.01; Lek et al., 2016) and evolutionary conserved (LINSIGHT score > 0.8; Huang et al., 2017). (B) Q-Q plot depicting on x axis the −log 10 of expected p value versus the actually measured p value for 3,761 enhancer groups using whole-genome sequencing (WGS) data from 4,495 ALS cases and 1,925 controls. *CAV1/CAV2* enhancers deviate from the null distribution (diagonal), revealing that the burden of variants measured in *CAV1/CAV2* enhancers is significantly associated with risk of ALS even after correction for multiple testing. (C) Quantity of genetic material (bp) relative to CNS enhancers derived from Hi-C data (Rhie et al., 2018); CNS enhancers = 100. Upper two bars denote total genetic material; lower two bars denote ALS-associated genetic variants only. Gray shading denotes material unique to CNS or tissue-agnostic enhancers versus material shared by both (white). (D and E) *CAV1*-coding variants passing filtering criteria are depicted in the table and figure. This analysis utilized WGS data from 4,495 ALS cases and 1,925 controls. One variant is present at higher frequency in controls (orange arrow), and one variant is present is multiple ALS patients (bold arrow); all other variants were discovered in a single ALS patient and zero controls.

Our pipeline for testing for disease-associated genetic variation within enhancers is detailed in Figure 1A. Following the aggregation of enhancers linked to individual coding genes, we filtered enhancer variants to remove those unlikely to be pathogenic prior to association testing. Enhancer variants were included if minor allele frequency (MAF) < 0.01 (Lek et al., 2016; van Rheenen et al., 2016) and LINSIGHT (Huang et al., 2017) score >0.8. LINSIGHT score >0.8 is consistent with strong evolutionary selection (Huang et al., 2017). Following filtering, case and control variant frequencies for each set of enhancers were collapsed into a single SKAT burden test (STAR Methods; Lee et al., 2012).

We tested our pipeline using whole-genome sequencing (WGS) data from 4,495 ALS cases and 1,925 controls (Project MinE; Data-Freeze-1). First, we hypothesized correctly that aggregated enhancers linked to all genes within the “amyotrophic lateral sclerosis” KEGG pathway (Kanehisa et al., 2017) would be enriched with ALS-associated genetic variation (SKAT-O; p = 0.02; 377 variants). Second, we hypothesized that pathogenic enhancer variants are likely to cause reduced transcription of their target coding gene, which might be expected to mimic a haploinsufficiency mechanism. Therefore, we examined variants within enhancers linked to expression of *TBK1*, which is uniquely known to cause ALS via haploinsufficiency (Freischmidt et al., 2015). Consistent with our hypothesis, genetic variation within *TBK1* enhancers is significantly associated with ALS (p = 0.003; SKAT-O; 12 variants; Table S1). Finally, expression of *TBK1* was reduced in patient-derived lymphoblastoid cells carrying an ALS-associated chr12:65059913G>A mutation within a TBK1 enhancer compared to mean expression in cells derived from neurologically normal controls, although this difference was not statistically significant (24% reduction; p = 0.27; Welch’s t test; Figure S2).

### Genetic Variation within *CAV1/CAV2* Enhancers Is Linked to ALS

We applied our pipeline to test for genetic association within enhancers linked to *CAV1* and *CAV2* expression. We discovered significant enrichment of ALS-associated genetic variation within enhancers linked to *CAV1* (p = 3.88 × 10^−5^; SKAT-O; 40 variants) and *CAV2* (p = 1.52 × 10^−5^; 57 variants). In total, 56 (1.2%) sporadic ALS patients carry one or more ALS-associated variants within *CAV1*/*CAV2* enhancers compared to 2 (0.1%) of controls (risk ratio = 12.0). There is significant overlap between enhancers and ALS-associated variants linked to *CAV1* and *CAV2* (Tables S1 and S3), which reflects shared function between the two proteins.

As a final test of our pipeline, we applied our analysis to all well-annotated genes found within KEGG pathways (n = 3,761). In this analysis, *CAV1* and *CAV2* enhancers carry the most significant enrichment with ALS-associated mutations compared to all other genes (Figure 1B). Importantly, there was no inflation of p values to indicate false positives (λGC 1,000 = 1.07).

### Genetic Variation within CAV1 CNS Enhancers Is Associated with ALS

To test whether ALS-associated genetic variation within *CAV1*/*CAV2* enhancers is relevant within the CNS, we re-tested for genetic association using CNS-specific enhancers. A recent study released Hi-C data from CNS neurons (Rhie et al., 2018). We used these data to recalculate enhancer-gene relationships for *CAV1* (Table S1); no data were available for *CAV2*. Despite a significant change in the number and location of aggregated variants (Figure 1C), genetic variation within *CAV1* CNS enhancers was still significantly associated with ALS (SKAT-O; p = 6.36E−05; 128 variants; Table S3); 188 (4.1%) ALS patients carried an ALS-associated *CAV1* CNS enhancer risk variant compared to 17 (0.9%) of controls (risk ratio = 4.6).

### Replication of ALS-Associated Genetic Variation within CAV1 and CAV2 Enhancers

To validate observed genetic association within *CAV1/CAV2* enhancers, we obtained WGS data from an independent cohort of 1,685 ALS patients and 445 controls (Project MinE; Data-Freeze-2). Derived tissue-agnostic *CAV1/CAV2* enhancers were not sufficiently variable in this smaller cohort (<10 variants), though Hi-C-derived CNS enhancers contained more genetic material. Re-applying our pipeline to *CAV1* Hi-C-derived CNS enhancers in the validation cohort revealed significant enrichment of ALS-associated genetic variation (p = 0.03; SKAT-O; 51 variants; Table S3); 60 (3.6%) ALS patients carried an ALS-associated *CAV1* CNS enhancer risk variant compared to 3 (0.7%) of controls (risk ratio = 5.1).

As a secondary analysis, we obtained summary statistics from 32,298 European non-Finnish non-ALS controls (Karczewski et al., 2020; Taliun et al., 2019). A larger sample size facilitated re-analysis of tissue-agnostic *CAV1/CAV2* enhancers within the same ALS cohort but using an independent population matched control cohort. Re-applying our pipeline revealed significant enrichment of ALS-associated genetic variants within *CAV1* and *CAV2* enhancers compared to the discovery cohort (n = 4,495, Data-Freeze-1; *CAV1*: p = 2.64 × 10^−9^, SKAT-O, 112 variants; *CAV2*: p = 7.30 × 10^−8^, SKAT-O, 174 variants) and replication cohort (n = 1,685, Data-Freeze-2; *CAV1*: p *=* 8.6 × 10^−5^, SKAT-O, 94 variants*; CAV2*: p *=* 4.87 × 10^−7^, SKAT-O, 150 variants)*.*

### Genetic Variation within *CAV1* Coding Sequence Is Associated with ALS

It is likely that genetic variation within linked enhancer and coding regions can produce similar phenotypes. We tested for ALS-associated genetic variation within *CAV1* and *CAV2* exons by rare-variant burden testing using WGS data from 4,495 ALS cases and 1,925 controls (Project MinE; Data-Freeze-1). In addition to filtering by MAF < 0.01 (van Rheenen et al., 2016), we introduced a functional filter to identify variants that alter protein function (STAR Methods; Cingolani et al., 2012). In *CAV1*, but not *CAV2*, coding sequence, we identified significant enrichment of functional genetic variation in ALS patients (p = 0.03; 12 variants; Firth logistic regression; beta = 1.47; Figures 1D and 1E). In total, 15 (0.3%) ALS patients carried a *CAV1* coding variant compared to 1 (0.05%) of controls (risk ratio = 6.4). Coding and enhancer mutations occurred in independent individuals, which excludes the possibility that the observed convergence is a consequence of linkage disequilibrium.

### Reduced *CAV1* and *CAV2* Expression in Patient-Derived Cells Carrying an ALS-Associated Enhancer Variant

Burden testing derives power from aggregating mutations into a single statistical test, but as a consequence, experimental evaluation is necessary to determine which individual mutations are pathogenic: indeed, it is likely that a significant proportion of variants are not pathogenic (Lee et al., 2012). Reduced CAV1 expression is toxic to neurons (Head et al., 2010, 2011), and therefore, we measured *CAV1/CAV2* expression in lymphoblastoid cells derived from ALS patients carrying *CAV1/CAV*2 enhancer variants: chr7:116222625T>C and chr7:115994269:C>T (Table S3).

**Figure 2:**
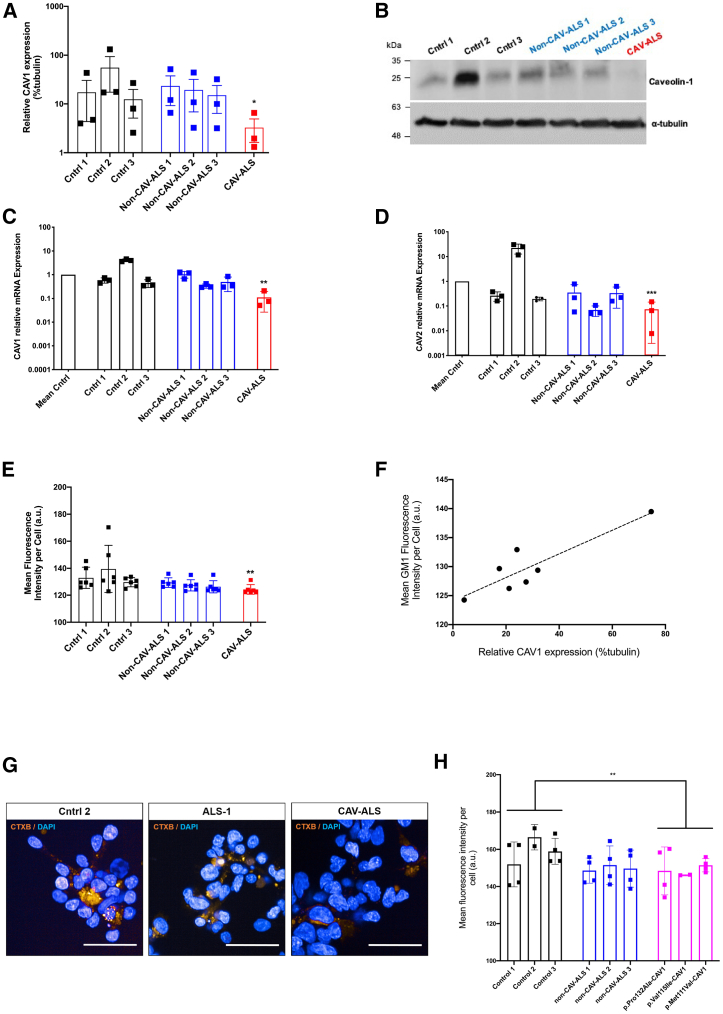
Patient-Derived Lymphoblastoid Cell Lines Carrying an ALS-Associated *CAV*-Enhancer/Coding Variants Have Reduced Expression of *CAV1*/*CAV2* and Disrupted MLR Lymphoblastoid cells were derived from neurologically normal controls (n = 3, black), ALS patients without *CAV*-enhancer variants (n = 3, blue), ALS patients carrying *CAV1*-coding mutations (n = 3, magenta), and cells carrying a disease-associated chr7:116222625T>C-*CAV1/CAV2* enhancer variant (red). (A and B) Immunoblotting measurement of CAV1 protein expression relative to α-tubulin loading control with an example blot. (C and D) qPCR measurement of *CAV1* and *CAV2* mRNA expression relative to mean expression in normal controls; expression normalized relative to loading control. (E, G, and H) Measurement of MLR integrity by live-cell imaging and GM1 labeling with CTxB. CTxB fluorescence is plotted with example images. Scale bar, 50 μm. (F) CAV1 protein expression is plotted versus MLR integrity as measured by CTxB fluorescence in the same cell line, with regression line (dotted). Data presented as mean ± 1 SD. ^∗^p < 0.05; ^∗∗^p < 0.01; ^∗∗∗^p < 0.001.

In cells carrying chr7:116222625T>C, CAV1 protein (89% reduction; p = 0.05; Mann-Whitney test; Figures 2A and 2B) and mRNA (89% reduction; p = 0.003; Welch’s t test; Figure 2C) and *CAV2* mRNA (93% reduction; p = 0.002; Welch’s t test; Figure 2D) expression was significantly reduced compared to mean expression in cells derived from neurologically normal controls. Unfortunately, immunoblotting for CAV2 was not possible due to lack of a sufficiently specific antibody. Expression was also reduced compared to ALS patients without an enhancer mutation (Figures 2A–2D).

Cells carrying chr7:115994269:C > T did not show reduced expression of *CAV1/CAV2* (data not shown). We speculate that this mutation may impact transcription only in CNS cells, or alternatively, this variant may be non-functional.

### Impaired MLR Formation in Patient-Derived Cells Carrying an ALS-Associated Enhancer Variant

Reduced CAV1/CAV2 function is proposed to be toxic via disruption of MLR, leading to impaired cell signaling (Sawada et al., 2019). We tested whether ALS-associated enhancer variants that reduce *CAV1/CAV2* expression also impair MLR formation. MLR integrity was measured by expression of GM1 gangliosides as labeled by cholera-toxin B (CTxB) (Aman et al., 2001; Day and Kenworthy, 2015; Sawada et al., 2019). CTxB fluorescence is significantly reduced in lymphoblastoid cells carrying chr7:116222625T>C compared to cells derived from neurologically normal controls (8% reduction; Figure 2E); fluorescence was also reduced compared to ALS patients without a *CAV1/CAV2* enhancer variant (Figures 2E and 2G). Strikingly, in all cell lines, GM1 expression and CAV1 protein expression are positively correlated (r = 0.89; p = 0.007; Pearson correlation; Figure 2F), which is consistent with direct dependence of MLR integrity on CAV1 function.

We hypothesized that *CAV1*-coding variants would produce a similar effect on MLR formation. Consistent with this, CTxB fluorescence is significantly reduced in lymphoblastoid cells carrying p.Met111Val-, p.Pro132Ala-, and p.Val155Ile-CAV1 mutations compared to cells derived from neurologically normal controls (p = 0.009; t test; Figure 2H).

### CRISPR-SpCas9 Enhancer Editing Reduces *CAV1/CAV2* Expression in Neurons

We have confirmed that patient-derived cells carrying an ALS-associated *CAV1/CAV2* enhancer mutation display reduced *CAV1/CAV2* expression and disrupted MLR, which is likely to lead to neurotoxicity (Sawada et al., 2019). However, these experiments were carried out in non-neuronal cells. To confirm that disruption of the same enhancer is sufficient to reduce *CAV1/CAV2* expression in a human CNS-relevant neuronal cell, we used CRISPR-SpCas9 editing to introduce indel mutations proximal to the site of the chr7:116222625T>C mutation in SH-SY5Y cells, which were subsequently differentiated into neurons.

**Figure 3:**
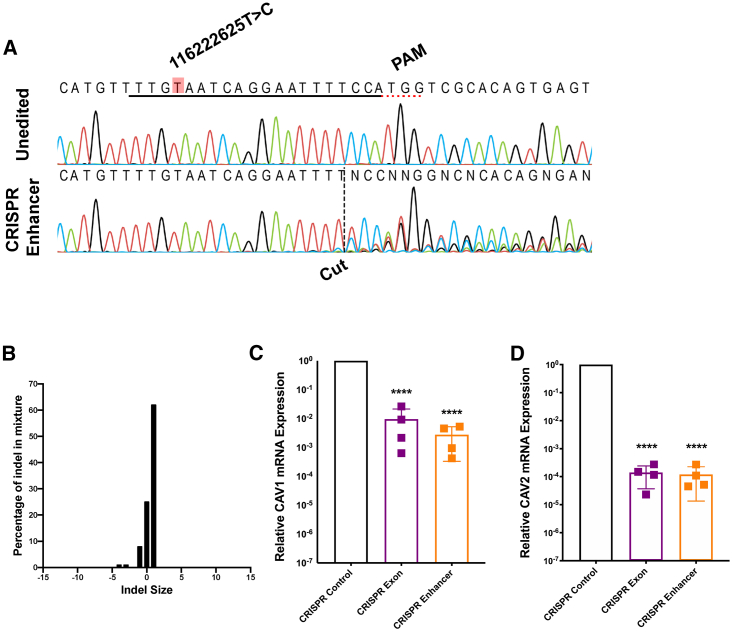
CRISPR-Directed Perturbation of a *CAV*-Enhancer Region Proximate to a Patient Mutation Reduces *CAV1*/*CAV2* Expression in a Differentiated SH-SY5Y Neuronal Cell (A) Sanger sequencing traces demonstrating spCas9 cut site adjacent to PAM and subsequent waveform decomposition in enhancer edited cells. Position of chr7:116222625T>C mutation is indicated. Black line indicates gRNA sequence. (B) Indel distribution within *CAV*-enhancer region in CRISPR-edited SH-SY5Y cells. (C and D) qPCR measurement of *CAV1* mRNA and *CAV2* mRNA reveals reduced expression in *CAV*-enhancer and *CAV1*-exon CRISPR-edited neurons compared to CRISPR editing of *HPRT*; expression normalized relative to loading control. Data presented as mean ± 1 SD. ^∗∗∗∗^p < 0.0001.

Guide RNAs (gRNAs) were designed to target a protospacer adjacent motif (PAM) site 16 bp downstream of the chr7:116222625T>C mutation site. Sanger sequencing and waveform decomposition analysis (Hsiau et al., 2018) revealed 72% editing efficiency in undifferentiated SH-SY5Y cells (Figure 3A). The majority of introduced changes were a single-nucleotide insertion (chr7:116222638T>TT; Figure 3B). A commercially available gRNA targeting *CAV1* exon 2 was chosen to introduce a nonsense mutation and served as a positive control, and a commercially available control gRNA targeting *HPRT* served as a negative control. CRISPR-SpCas9-edited SH-SY5Y cells were differentiated to a neuronal phenotype; successful differentiation was confirmed by altered expression of PAX6 (Figure S4A; Forster et al., 2016) and increased total dendritic length (p = 0.01; paired t test; Figure S4B; Forster et al., 2016). Differentiated cells were harvested, and RNA was extracted for qPCR. We confirmed reduced expression of *CAV1* (>99% reduction; p < 0.0001; Welch’s t test) and *CAV2* (>99% reduction; p < 0.0001) mRNA in enhancer edited cells (Figures 3C and 3D). *CAV2* mRNA expression was reduced in the context of enhancer editing but also by *CAV1*-coding editing, which likely reflects interdependence between the two genes (Drab et al., 2001). Extreme reductions in *CAV1/CAV2* expression are notable; however, phenotypic change in excess of editing efficiency is well described and may be a consequence of CRISPR interference (Gaj et al., 2017).

### ALS-Associated *CAV1/CAV2* Enhancer Mutation Is Associated with TF Binding Sites

Pathogenic enhancer mutations may act via altered TF binding (Karnuta and Scacheri, 2018). To identify potential changes in TF binding associated with *CAV1/CAV2* enhancer mutations, we analyzed publicly available ChIP sequencing data (https://genome.ucsc.edu; TF ChIP-seq Clusters from ENCODE 3, version: 3 November 2018). We identified five DNA-binding proteins associated with both the site of the chr7:116222625T>C mutation and the cut site for our CRISPR-SpCas9-editing experiment in at least one cell type: RAD21; CTCF; FOS; SMC3; and CEBPB. However, introduced mutations did not significantly alter the predicted strength of TF binding (STAR Methods; Nguyen et al., 2018).

## Discussion

Genetic discoveries in ALS have focused on high effect variants within coding genes in patients with autosomal dominant inheritance. The non-coding genome is thought to contain missing heritability (Pallares, 2019). We developed an approach to discover genetic association within gene enhancer elements. Using our methodology, we successfully identified and validated ALS-associated genetic variation within enhancer and coding regions associated with *CAV1*; we therefore propose *CAV1* to be an ALS risk gene. It is notable that we did not identify ALS-associated nonsense mutations within *CAV1* or *CAV2*. *CAV1/CAV2* nonsense mutations would dramatically reduce gene expression irrespective of cell type or even developmental stage and may manifest a broad range of phenotypes, including motor neuron toxicity, but not specifically ALS. A traditional focus only on loss-of-function coding variants may therefore have missed the link between *CAV1/CAV2* expression and ALS.

Our work builds upon previous observations that CAV1 function is neuroprotective in neurodegenerative disease (Head et al., 2010) and in ALS in particular (Sawada et al., 2019). Until now, it was not clear whether CAV1 dysfunction was a cause or effect of neuronal toxicity; our discovery of genetic risk associated with *CAV1/CAV2* expression places this pathway upstream in the development of disease. Using patient-derived cells, we have shown that ALS-associated genetic variation within *CAV1/CAV2* enhancers and *CAV1* coding sequence reduces *CAV1/CAV2* expression and disrupts MLR, which is contingent with impaired neurotrophic signaling and consequently neurodegeneration (Sawada et al., 2019). Moreover, CRISPR-SpCas9 perturbation proximate to a *CAV/CAV2* enhancer mutation reduced *CAV1* and *CAV2* expression in human neuronal cells, suggesting that this enhancer region is functional within the CNS.

Enhancer function is thought to depend on the binding of TFs (Koch et al., 2011). Current understanding of function within enhancer regions is limited (Levo et al., 2015), in part because of a paucity of variants with validated biological impact. This incomplete understanding is reflected in our failure to link a change in TF binding to the changes in *CAV1/CAV2* expression we observe. Our discovery forms a platform for improved understanding of molecular function within these regions. We propose that our approach, namely the discovery of disease-associated genetic variation, is a means of overcoming a reliance on unphysiological *in vitro* assays to understand enhancer biology (Gasperini et al., 2020).

Our genetic study was performed in sporadic ALS cases. As a result, we propose that the mutations we have identified are likely to be risk factors rather than fully penetrant monogenic causes of disease. Indeed, sporadic ALS is proposed to be a multistep process involving both genetic and environmental insults (Al-Chalabi et al., 2014). The association of CAV1 function with neurotrophic signaling is consistent with this premise; identified deficient neurotrophic signaling in ALS has been proposed as a risk factor that increases the vulnerability of motor neurons to additional insults (Bemelmans et al., 2006; Ghavami et al., 2014; Kowiański et al., 2018; Tooze and Schiavo, 2008).

The premise of personalized medicine for complex disease is that gene-environment interactions leading to disease are likely to be heterogeneous (Li et al., 2019). We suggest that, in a significant number of ALS patients, genetic mutations leading to reduced CAV1/CAV2 function are a significant cause of neuronal toxicity. Upregulation of CAV1 is in development as a therapeutic tool (Head et al., 2011; US patent no. 8969077B2); our data suggest that this could be applied to genetically selected ALS patients in a personalized medicine approach.

## STAR★Methods

### Key Resources Table

**Table:** 

REAGENT or RESOURCE	SOURCE	IDENTIFIER
**Antibodies**		

Anti-Caveolin-1	GeneTex	#GTX100205; RRID:AB_1240559
Anti-Caveolin-1	Abcam	#AB2910; RRID:AB_303405
α-tubulin	Sigma	#T9026; RRID:AB_477593
Anti-Pax6	Abcam	#AB5790; RRID:AB_305110
Anti-mouse HRP-conjugate	Promega	#W4021; RRID:AB_430834
Anti-rabbit HRP-conjugate	Promega	#W4011; RRID:AB_430833
Donkey anti-rabbit Alexa568 secondary	Invitrogen	#A10042; RRID:AB_2534017
Donkey anti-mouse Alexa488 secondary	Invitrogen	#A-21202; RRID:AB_141607

**Chemicals, Peptides and Recombinant Proteins**		

Alt-R® S.p. Cas9 Nuclease V3	Integrated DNA technologies	#1081059
Alt-R® Cas9 Electroporation Enhancer	Integrated DNA technologies	#1075915
Dulbecco’s Modified Eagle medium	Lonza	#12-604F
RPMI-1640 medium with L-glutamine	Lonza	#BE12-702F
Neurobasal medium	ThermoFisher Scientific	#12348017
Penicillin-Streptomycin	Sigma	#P4333
Fibronectin	Merck	#FC010
10x Trypsin	Sigma	#59427C
Foetal bovine serum	ThermoFisher Scientific	#10270106
L-glutamine (200mM)	ThermoFisher Scientific	#25030081
Agarose	Melford	#MB1200
Ethidium bromide solution	Sigma	#E1510
VeriFi mix red	PCRBio	#PB10.42-01
Tri reagent	Sigma	#93289-100ML
M-MLV reverse transcriptase	ThermoFisher Scientific	#28025-013
5x First Strand buffer	ThermoFisher Scientific	#18057-018
0.1M Dithiothreitol	ThermoFisher Scientific	#707265ML
dNTP Mix	ThermoFisher Scientific	#10534823
SYBR Green Brilliant III master mix	Agilent	#600882
Random hexamer primer	ThermoFisher Scientific	#SO142
Pre-stained protein ladder	Abcam	#116028
Bradford reagent	Bio-Rad	#5000001
Laemmli buffer	Bio-Rad	#1610747
b-mercaptoethanol	Sigma	#M6250
EDTA	Sigma	#E5134
HEPES	Sigma	#H3375
SigmaFAST Protease Inhibitor Cocktail tablets	Sigma	#S8820
PMSF protease inhibitor	ThermoFisher Scientific	#36978
Clarity Western ECL blotting substrate	Bio-Rad	#1705060S
TracrRNA	Integrated DNA technologies	#1072533
TE Buffer, RNase-free pH 8	ThermoFisher Scientific	#AM9849
Dulbecco’s Phosphate Buffered Saline	Sigma	#D8537-500ML
Triton X-100	Sigma-Aldrich	#T8787
Normal horse serum	Vector	#S-2000-20
Hoechst 33342	ThermoFisher Scientific	#62249
All-trans retinoic acid	Sigma	#R2625
BDNF	PeproTech	#450-02
N-2 supplement	ThermoFisher Scientific	#17502048
Cholera Toxin Subunit B (recombinant) Alexa555 Conjugate	Invitrogen	#C22843

**Critical Commercial Assays**		

Pierce BCA Assay Protein Assay Kit	ThermoFisher Scientific	#23225
GenElute Mammalian Genomic DNA Miniprep Kit	Sigma	#G1N350
Direct-zol RNA Miniprep Kit	Zymo Research	#R2050
Neon Transfection System 10 μL Kit	ThermoFisher Scientific	#MPK1096
Alt-R CRISPR-Cas9 Control Kit, Human, 2 nmol	Integrated DNA technologies	#1072554

**Experimental Models: Cell Lines**		

SH-SY5Y	ATCC	Cat.#CRL-2266
Patient and control LCL lines	MNDA (UK) DNA Bank	N/A

**Oligonucleotides**		

See Supplementary Table S5		N/A

**Software and Algorithms**		

SKAT	https://cran.r-project.org/web/packages/SKAT/index.html	N/A
R	https://cran.r-project.org/mirrors.html	N/A
Galaxy	https://usegalaxy.org/	N/A
snpStats	https://www.bioconductor.org/packages/release/bioc/html/snpStats.html	N/A
VariantAnnotation	https://www.bioconductor.org/packages/release/bioc/html/VariantAnnotation.html	N/A
Plink	http://zzz.bwh.harvard.edu/plink/download.shtml	N/A
PRISM 7	GraphPad	N/A
ICE CRISPR analysis tool	https://ice.synthego.com/#/	N/A
CRISPOR guide RNA design tool	http://crispor.tefor.net/	N/A
CFX Maestro	Bio-Rad	N/A
Harmony High-Content Imaging and Analysis Software	PerkinElmer	N/A
FIJI (FIJI Is Just ImageJ)	NIH	N/A

### Resource Availability

#### Lead Contact

Further information and requests for resources and reagents should be directed to and will be fulfilled by the corresponding author, Johnathan Cooper-Knock (j.cooper-knock@sheffield.ac.uk).

#### Materials Availability

All unique/stable reagents generated in this study are available from the Lead Contact without restriction.

#### Data and Code Availability

This study did not generate any unique datasets or code. Whole genome sequencing data is available through Project MinE (https://www.projectmine.com/research/data-sharing/). A data access committee controls access to raw data, ensuring a FAIR data setup (https://www.datafairport.org).

### Experimental Model and Subject Details

#### Selection of patients and controls for genetic sequencing

All 6,180 patients and 2,370 controls included in this study were recruited at specialized neuromuscular centers in the UK, Belgium, Germany, Ireland, Italy, Spain, Turkey, the United States and the Netherlands (Project MinE ALS Sequencing Consortium, 2018). Patients were diagnosed with possible, probable or definite ALS according to the 1994 El-Escorial criteria (Brooks, 1994). All controls were free of neuromuscular diseases and matched for age, sex and geographical location. Analysis focused on *Data-Freeze-1* including 4,495 ALS patients and 1,925 controls; *Data-Freeze-2* (released December 2019) was used for validation. After excluding population outliers *Data-Freeze-2* included 1,685 ALS patients and 445 controls.

Secondary analysis was performed using summary statistics derived from WGS of 32,298 European non-Finnish controls. This cohort and the relevant analysis pipeline have been previously described (Karczewski et al., 2020).

The study was approved by the South Sheffield Research Ethics Committee. Also, this study followed study protocols approved by Medical Ethical Committees for each of the participating institutions. Written informed consent was obtained from all participating individuals. All methods were performed in accordance with relevant national and international guidelines and regulations.

#### SH-SY5Y neuroblastoma cells

Human SH-SY5Y neuroblastoma cells were cultured in Dulbecco’s Modified Eagle’s Medium (DMEM) (Lonza) supplemented with 10% (v/v) fetal bovine serum (FBS) (Thermo-Fisher Scientific), 50 units/mL of penicillin and 50 μg/mL of streptomycin. Cell lines were maintained at 5% CO2 in a 37°C incubator and split every 3-4 days. All experimental work was performed using cells within the range of 7-32 passages.

#### Patient-derived lymphoblastoid cells

Lymphoblastoid cell lines derived from Caucasian ALS patients (n = 9) and neurologically normal controls (n = 3), all of Northern European descent, were obtained from the UK Motor Neurone Disease Association (MNDA) DNA Bank. Demographic details are provided in Table S6. Lymphoblastoid cells were cultured in RPMI 1640 Medium (Lonza) supplemented with 2mM L-glutamine and 10% (v/v) FBS. Cells were maintained at 5% CO_2_ in a 37°C incubator and split every 3-4 days.

### Method Details

#### High Throughput DNA sequencing and QC

Methods are described elsewhere (Project MinE ALS Sequencing Consortium, 2018). In brief, DNA was extracted from venous blood samples and quality was assessed by gel electrophoresis. DNA samples were sequenced using Illumina’s FastTrack services (San Diego, CA, USA) on the Illumina HiSeq 2000 platform. Sequencing was 100 bp paired-end (~40 × coverage) performed using PCR-free library preparation. The Isaac pipeline (Raczy et al., 2013) was used for alignment to the hg19 reference genome as well as to call single nucleotide variants (SNVs), insertions and deletions (indels), and larger structural variants (SVs). Variants not passing Isaac’s quality filter were set to missing; non-autosomal chromosome and multi-allelic variants were excluded. Sample and SNP QC were performed using PLINK (Chang et al., 2015; Purcell et al., 2007) and VCFtools (Danecek et al., 2011). Samples were excluded if missingness by sample < 10% across all 22 chromosomes. Remaining sample QC steps were performed on a set of high-quality biallelic SNPs that had minor allele frequency (MAF) > 10%, missingness < 0.1%, were LD-pruned at an r^2^ threshold of 0.2, were not A/T or C/G SNPs, did not lie in the major histocompatibility complex (MHC) or LCT locus, and did not occur in the inversions on chromosome 8 or chromosome 17. The ~30,000 SNPs overlapping this set of SNPs and HapMap 3 (HM3) were used to calculate principal components projecting the ALS cases and controls onto the HM3 samples. Samples of non-European ancestry, defined as further than 10 standard deviations from the European-ancestry populations in HM3, were excluded from further analysis. Samples with an inbreeding coefficient > 3 s.d. from the mean of the distribution were excluded, as were unexpected related samples. Samples with discordant sex information (comparing chromosome X genotypes and phenotype information) were excluded.

Variants with missingness > 5% were removed, as were variants out of Hardy-Weinberg equilibrium in controls (p < 1 × 10^−6^) and monomorphic variants (induced by sample exclusions). Differential missingness between cases and controls was checked and variants with p < 1 × 10^−6^ were removed. Variants with extreme depth of coverage (> 6 s.d. from the mean of the total depth distribution) were also excluded. Finally, the mitochondrial, X and Y chromosomes were excluded from analysis. Approximately 10 million sites were lost during variant QC.

#### Variant Filtering

ALS features a polygenic rare variant architecture (van Rheenen et al., 2016); therefore all searches for pathogenic variants in enhancer and coding regions featured a filter for MAF within the Genome Aggregation Database (gnomAD) of < 1/100 control alleles (Lek et al., 2016). Additional filtering varied between area reflecting differences in function. In enhancer regions variants were included only if evolutionary conserved based on a LINSIGHT score > 0.8 (Huang et al., 2017). In coding regions we filtered for variants with impact on protein function as defined by snpeff (Cingolani et al., 2012): Variants annotated HIGH/MODERATE/LOW impact were included, but we excluded variants annotated ‘synonymous’ or ‘TF_binding_site_variant’ because these functions are independent of amino acid sequence.

#### Cell lysis

Lymphoblastoid cells were lysed in urea lysis buffer [8M urea; 1% (w/v) DTT; 20% (w/v) SDS; 1.5M Tris pH 6.8; + dH_2_O) + PIC (20μL/mL) + 1mM PMSF at room temperature (RT). Samples were sonicated at 50% amplitude for 10 s (SoniPrep 150, MSE) followed by a 30 s incubation at RT (3x). Samples were then incubated at RT for 15 minutes. Lysates were centrifuged at 17,000xg for 5 minutes at RT. Total protein concentration within the supernatant was quantified using a Pierce BCA Protein Assay Kit (ThermoFisher Scientific) according to the manufacturer’s instructions and absorbance was measured at 562nm on a PHERAstar FS spectrophotometer (BMG Biotech). Lysates were mixed with 4x Laemmli buffer (277.8mM Tris-HCl; 44.4% (v/v) glycerol; 4.4% SDS; 0.02% bromophenol blue; 355mM 2-mercaptoethanol; pH 6.8) and boiled at 95°C for 5 minutes. Protein extracts were fractionated on 12% SDS polyacrylamide gels and electrophoretically transferred to nitrocellulose membranes.

#### Immunoblotting

Nitrocellulose membranes were initially blocked in 5% (w/v) milk (Marvel)/Tris Buffered Saline, with Tween® 20 (TBST) (20mM Tris, 137mM NaCl, 0.2% (v/v) Tween® 20, pH 7.6) for 1 hour at RT then probed using the relevant primary antibody overnight at 4°C. Anti-caveolin 1 (GeneTex) (1:500 dilution) and α-tubulin (Abcam) (1:2000 dilution) primary antibodies were detected using a horseradish peroxidase (HRP)-conjugated rabbit secondary antibody and a HRP-conjugated mouse secondary antibody, respectively (Promega) (1:5000 dilution). Protein bands were visualized using ECL substrate (Bio-Rad) and the chemiluminescence signal was imaged using a G:BOX (Syngene).

#### CRISPR editing of mammalian cell lines

Guide RNAs (gRNAs) were designed using the Crispor tool (http://crispor.tefor.net/) to target CAV-enhancer regions. Design was guided by proximity to patient enhancer mutation sites, available protospacer adjacent motifs (PAM), and predicted on- and off- target efficiencies. gRNAs targeting within 30bp either side of the patient enhancer mutation site (chr7:116222625, hg19) were considered and screened for editing efficiency as below. One guide sequence (5′ -UUGUAAUCAGGAAUUUUCCA-3′) was most efficient and chosen for subsequent experimentation. Validated, commercially available CRISPR control targeting HPRT (IDT) and CAV1 exon-targeting (ThermoFisher Scientific) gRNAs were also obtained (Table S5). gRNA duplexes were assembled from tracrRNA and crRNA in a thermocycler according to manufacturer’s instructions under RNase-free conditions. Cells were cultured to ensure 70%–90% confluency on the day of transfection. 1ml antibiotic-free DMEM (Lonza) was prepared and incubated in 24-well plates at 37°C. CRISPR/Cas9 Ribonucleoproteins were formed by complexing 240ng gRNA duplex with 1250ng Alt-R V3 Cas9 Protein (IDT) in 10 μL buffer R (from 10 μL Neon transfection kit, ThermoFisher Scientific) - a 1:1 molar ratio - for 10 minutes. 100,000 viable cells were aliquoted per transfection and centrifuged at 400 x g for 4 minutes. Cells were washed in calcium- and magnesium-free Dulbecco’s Phosphate Buffered Saline (Sigma) and centrifuged at 400 x g for 4 minutes. Cell pellets were resuspended in 10 μL buffer R containing Cas9 protein and gRNA duplexes. 2 μL of 10.8 μM electroporation enhancer (IDT) was added and the solution mixed thoroughly to ensure a suspension of single cells. 10 μL of this mixture was loaded into a Neon transfection system (ThermoFisher Scientific) and electroporated according to manufacturer’s instructions (1200V, 3 pulse, 20 s pulse width for SH-SY5Y cells). Cells were then transferred to pre-warmed media in 24-well plates.

#### Determining CRISPR editing efficiency

Genomic DNA was isolated from CRISPR-edited and control cells using a GenElute Mammalian DNA Miniprep Kit (Sigma) according to manufacturer’s instructions. A ~400bp region around the expected cas9 cut site was amplified by polymerase chain reaction using VeriFi mix (PCRbio). Expected amplification was confirmed using gel electrophoresis, and the products were Sanger-sequenced. Sequencing trace files were uploaded to ICE (https://ice.synthego.com) and an indel efficiency calculated.

#### Quantitative PCR (RT-PCR):

Cells were cultured until at least 70% confluent, lysed on ice using an appropriate volume of Tri Reagent (Sigma) for 5 minutes and transferred to 1.5ml RNase-free tubes. Total RNA was extracted using a Direct-zol RNA Miniprep Kit (Zymo) according to manufacturer’s instructions, and RNA concentration confirmed using a NanoDrop spectrophotometer (ThermoFisher Scientific). 2 μg of total RNA was then converted to cDNA by adding 1 μL 10mM dNTPs, 1 μL 40 μM random hexamer primer (ThermoFisher Scientific), and DNase/RNase-free water to a total volume of 14 μL. This mixture was heated for 5 minutes at 70°C then placed on ice for 5 minutes. 4 μL of 5x FS buffer, 2 μL 0.1M DTT, and 1 μL M-MLV reverse transcriptase (ThermoFisher Scientific) were then added and cDNA conversion performed in a PCR thermocycler (37°C for 50 minutes, 70°C for 10 minutes). cDNA was amplified using RT-PCR with Brilliant III SYBR Green (Agilent) as per manufacturer’s instructions. Ct analysis was performed using CFX Maestro software (BioRad). Reference genes RPL13A and GAPDH were chosen for experiments involving lymphoblast cells and SH-SY5Y cells respectively, for their relative stability between experiments in these cell lines (Hoerndli et al., 2004; Hruz et al., 2011). Relative mRNA expression values were then calculated using the 2^-ΔΔCT^ method (Schmittgen and Livak, 2008). Low CAV2 expression leading to non-amplification in one cell line was assigned a maximum CT value of 40 for one repeat (McCall et al., 2014).

#### SH-SY5Y neuronal differentiation

Human SH-SY5Y neuroblastoma cells were seeded at densities of either 5x10^4^ cells per well of a 6-well culture plate, or 2x10^3^ cells per well of a 96-well culture plate in DMEM (Lonza) supplemented with 10% (v/v) FBS, 50 units/mL penicillin and 50 μg/mL of streptomycin. 24 hours after seeding the media was changed to DMEM supplemented with 5% (v/v) FBS, 50 units/mL penicillin, 50 μg/mL of streptomycin, 4mM l-glutamine and 10 μM retinoic acid. After 72 hours, the medium was switched to neurobasal media (ThermoFisher Scientific) containing 1% (v/v) N-2 supplement 100x, 50 units/mL penicillin, 50 μg/mL of streptomycin, 1% l-glutamine and 50ng/mL human BDNF. Cells were cultured for an additional 3 days until fully differentiated.

To confirm neuronal differentiation blinded, semi-automated analysis of neurite length was performed using the SimpleNeuriteTracer plugin for FIJI (Longair et al., 2011). 2D images were converted to 8-bit grayscale and successive points along the midline of a neural process were selected. The software automatically identified the path between the two points. Tracing accuracy was improved using Hessian-based analysis of image curvatures. The AnalyzeSkeleton plugin (Arganda-Carreras et al., 2010) was used to quantify the morphology of the traces.

#### Immunocytochemistry

SH-SY5Y cells were fixed with 4% paraformaldehyde for 15 minutes and washed 3x with PBS. Cells were blocked in 5% normal horse serum containing 0.1% Triton X-100 for 1 hour at RT. All primary antibodies were diluted in blocking solution (anti-Caveolin-1, 1:500; α-tubulin, 1:2000; anti-Pax6, 1:200). Cells were incubated in the primary antibody for 2 hours at RT and washed 3x in PBS before incubation in the appropriate secondary antibody (1:1000 in PBS) for 1 hour at RT. Nuclear counterstain (Hoechst 33342) was applied for 10 minutes followed by a 3x wash in PBS. Cells were imaged using an Opera Phenix High Content Screening System (PerkinElmer).

#### Live cell imaging

96-well culture plates were coated in plasma fibronectin (Merck) (5μg/mL in PBS) for 30 minutes prior to cell seeding. Excess fibronectin was removed immediately before seeding lymphoblastoid cells at a density of 2x10^4^ cells per well. Lymphoblastoid cells were cultured in supplemented RPMI media for 2 days prior to live-cell imaging. Media was removed and 5μg/mL labeling solution (Cholera Toxin Subunit B [CTxB] + Hanks Balanced Salt Solution [HBSS]) was added to cell-containing wells and incubated for 45 minutes at 37°C, 5% CO_2_. Nuclear counterstain (Hoechst 33342) was applied for the final 5 minutes of the incubation. The labeling solution was removed, cells were washed 2x in PBS and incubated in 200μL pre-warmed HBSS for imaging. Live imaging was performed via confocal microscopy using an Opera Phenix™ High-Content Screening System (PerkinElmer) at 37°C, 5% CO_2_. Cells were visualized at 40x magnification within a high-resolution z stack consisting of images at 0.5μm intervals through the entire nuclear volume of the cell.

### Quantification and Statistical Analysis

#### Immunoblotting and quantitative PCR

Statistical analysis was conducted in GraphPad Prism 7 (La Jolla, CA). All bar graphs show the mean ± SD. To identify statistical differences between treatment groups utilized Welch’s unpaired t test.

#### Burden testing

The optimal unified test (SKAT-O) was used to perform burden testing in enhancer regions because it is optimized for large numbers of samples and for regions where a significant number of variants may not be causal (Lee et al., 2012). SKAT tests upweight significance of rare variants according to a beta density function of MAF in which *w*_*j*_ = *Beta*(*p*_*j*_, *a*_1_, *a*_2_), where *p*_*j*_ is the estimated MAF for SNP_*j*_ using all cases and controls, and the parameters *a*_1_ and *a*_2_ are prespecified. Optimal values of *a*_1_ and *a*_2_ were chosen using TBK1 enhancers where it was hypothesized ALS-associated should be present. Increasing values of *a*_2_ correspond to a relative upweighting of increasingly rare variants; optimum ALS-association was discovered for *a*_2_ = 250 (*a*_2_ = 25, p = 0.2; *a*_2_ = 250, p = 0.003; *a*_2_ = 2500, p = 0.01); therefore *a*_2_ = 250 was chosen for all further statistical tests.

When variants are expected to have equivalent functional impact SKAT can lose power (Basu and Pan, 2011) and therefore in coding regions where variant filtering was more stringent and based on functional as well as population/evolution observations Firth logistic regression was preferred. To adjust for confounders including population structure, burden testing used the first ten eigenvectors generated by principal components analysis of common variant profiles, and sex as covariates.

For the secondary analysis utilizing 32,298 European non-Finnish controls rare-variant burden testing was applied as before, except that sex and eigenvectors were not available for use as covariates.

#### Modeling of TF binding

Candidate TF were identified from ChIP-sequencing data clusters via the UCSC genome browser (https://genome.ucsc.edu track: ‘Transcription Factor ChIP-seq Clusters from ENCODE 3, version: 3 Nov 2018’). Clusters associated with chr7:116222625T > C were first identified, and then cross-referenced with other ALS-associated CAV enhancer variants (Table S3) and common TF identified. Changes in putative TF-binding capacity between wild-type, mutant and CRISPR/SpCas9-edited sequences associated with the chr7:116222625T > C mutation were then identified using position specific scoring matrices (http://rsat.sb-roscoff.fr/matrix-scan-quick_form.cgi ENCODE human TFs 2018 03: ‘CEBPB_disc1’, ‘RAD21_disc1’, ‘CTCF_disc1’). Relative weights for each TF/sequence combination were compared.

## Consortia

Project MinE ALS Sequencing consortium includes Ian Blair, Naomi Wray, Matthew Kiernan, Miguel Mitne Neto, Adriano Chio, Ruben Cauchi, Wim Robberecht, Philip van Damme, Phillippe Corcia, Phillipe Couratier, Orla Hardiman, Russel McLaughlin, Marc Gotkine, Vivan Drory, Nicola Ticozzi, Vincenzo Silani, Jan Veldink, Leonard van den Berg, Mamede de Carvalho, Jesus Mora Pardina, Monica Povedano, Peter Andersen, Markus Wber, Nazli Başak, Ammar Al-Chalabi, Christopher Shaw, Pamela Shaw, Karen Morrison, John Landers, and Jonathan Glass.

## Supplemental Information

Document S1. Figures S1 and S2 and Tables S1–S4

Document S2. Article plus Supplemental Information
