# Rare variant burden analysis within enhancers identifies *CAV1* as an ALS risk gene

**DOI:** 10.1016/j.celrep.2021.108730

**Published:** 2021-02-02

**Authors:** Johnathan Cooper-Knock, Sai Zhang, Kevin P. Kenna, Tobias Moll, John P. Franklin, Samantha Allen, Helia Ghahremani Nezhad, Alfredo Iacoangeli, Nancy Y. Yacovzada, Chen Eitan, Eran Hornstein, Eran Elhaik, Petra Celadova, Daniel Bose, Sali Farhan, Simon Fishilevich, Doron Lancet, Karen E. Morrison, Christopher E. Shaw, Ammar Al-Chalabi, Project MinE ALS Sequencing Consortium, Jan H. Veldink, Janine Kirby, Michael P. Snyder, Pamela J. Shaw

(Cell Reports *33*, 108456-1–108456-8.e1–e5; December 1, 2020)

In the originally published version of this article, Eran Elhaik was incorrectly spelled in the author list. The corrected author list appears here and with the article online.

The authors regret the error.

