# Supplementary material for: Rare Variant Burden Analysis within Enhancers Identifies CAV1 as an ALS Risk Gene
Source: Cell Rep. 2020 Dec 1;33(9):108456. doi: 10.1016/j.celrep.2020.108456 (PMC7710676; doi:10.1016/j.celrep.2020.108456)
Supplement: Document S1. Figures S1 and S2 and Tables S1–S4 [file mmc1.pdf]

Cell Reports, Volume *33*

Supplemental Information

Rare Variant Burden Analysis within Enhancers

Identifies *CAV1* as an ALS Risk Gene

Johnathan Cooper-Knock, Sai Zhang, Kevin P. Kenna, Tobias Moll, John P. Franklin, Samantha Allen, Helia Ghahremani Nezhad, Alfredo Iacoangeli, Nancy Y. Yacovzada, Chen Eitan, Eran Hornstein, Eran Ehilak, Petra Celadova, Daniel Bose, Sali Farhan, Simon Fishilevich, Doron Lancet, Karen E. Morrison, Christopher E. Shaw, Ammar Al-Chalabi, Project MinE ALS Sequencing Consortium, Jan H. Veldink, Janine Kirby, Michael P. Snyder, and Pamela J. Shaw

Cell Reports, Volume *33*

Supplemental Information

Rare Variant Burden Analysis within Enhancers Identifies *CAV1* as an ALS Risk Gene

Johnathan Cooper-Knock, Sai Zhang, Kevin P. Kenna, Tobias Moll, John P. Franklin, Samantha Allen, Helia Ghahremani Nezhad, Alfredo Iacoangeli, Nancy Y. Yacovzada, Chen Eitan, Eran Hornstein, Eran Ehilak, Petra Celadova, Daniel Bose, Sali Farhan, Simon Fishilevich, Doron Lancet, Karen E. Morrison, Christopher E. Shaw, Ammar Al-Chalabi, Project MinE ALS Sequencing Consortium, Jan H. Veldink, Janine Kirby, Michael P. Snyder, and Pamela J. Shaw

Table S1: Enhancer regions associated with CAV1/CAV2 or TBK1 expression in different tissues. Related to Figure 1

Gene Target	Chromosome	Start	Finish	Tissue
CAV1	chr7	116474804	116476617	Tissue-agnostic
CAV1	chr7	116057349	116058637	Tissue-agnostic
CAV1:CAV2	chr7	116202174	116202561	Tissue-agnostic
CAV1:CAV2	chr7	115898310	115902716	Tissue-agnostic
CAV1:CAV2	chr7	116215990	116219157	Tissue-agnostic
CAV1:CAV2	chr7	115992110	115999387	Tissue-agnostic
CAV1:CAV2	chr7	116185994	116188883	Tissue-agnostic
CAV1:CAV2	chr7	116209195	116214933	Tissue-agnostic
CAV1:CAV2	chr7	116196622	116201351	Tissue-agnostic
CAV1:CAV2	chr7	116149154	116154310	Tissue-agnostic
CAV1:CAV2	chr7	116220723	116224531	Tissue-agnostic
CAV1:CAV2	chr7	116033656	116036708	Tissue-agnostic
CAV2	chr7	116078147	116088487	Tissue-agnostic
CAV2	chr7	115954125	115959325	Tissue-agnostic
CAV2	chr7	116180927	116184847	Tissue-agnostic
CAV2	chr7	116231455	116233866	Tissue-agnostic
CAV2	chr7	116065132	116068292	Tissue-agnostic
CAV2	chr7	116094877	116096147	Tissue-agnostic
CAV2	chr7	116003024	116004444	Tissue-agnostic
CAV2	chr7	116071887	116076515	Tissue-agnostic
CAV2	chr7	116176051	116176914	Tissue-agnostic
CAV2	chr7	116261779	116262057	Tissue-agnostic
CAV2	chr7	116062851	116064462	Tissue-agnostic
CAV2	chr7	116134797	116136789	Tissue-agnostic
CAV2	chr7	116154567	116155823	Tissue-agnostic
CAV2	chr7	116104521	116107038	Tissue-agnostic
CAV1	chr7	115816693	115817206	CNS
CAV1	chr7	115853719	115854235	CNS
CAV1	chr7	115870180	115870885	CNS
CAV1	chr7	115871349	115873794	CNS
CAV1	chr7	115910552	115913260	CNS
CAV1	chr7	115913518	115914249	CNS
CAV1	chr7	115955578	115958367	CNS
CAV1	chr7	115967434	115969066	CNS
CAV1	chr7	115993415	115996411	CNS
CAV1	chr7	116034665	116036373	CNS
CAV1	chr7	116066587	116067242	CNS
CAV1	chr7	116073579	116076380	CNS
CAV1	chr7	116080916	116084930	CNS
CAV1	chr7	116094659	116096095	CNS
CAV1	chr7	116103858	116107331	CNS
CAV1	chr7	116118084	116119473	CNS
CAV1	chr7	116149961	116153401	CNS
CAV1	chr7	115898864	115900695	CNS

CAV1	chr7	115867068	115868903	CNS
CAV1	chr7	115996955	115998734	CNS
CAV1	chr7	116170083	116171009	CNS
CAV1	chr7	116175367	116176059	CNS
CAV1	chr7	116181441	116182810	CNS
CAV1	chr7	116182954	116183855	CNS
CAV1	chr7	116186806	116188332	CNS
CAV1	chr7	116195961	116196301	CNS
CAV1	chr7	116199565	116200132	CNS
CAV1	chr7	116206178	116207412	CNS
CAV1	chr7	116213507	116219235	CNS
CAV1	chr7	116221274	116225513	CNS
CAV1	chr7	116207998	116213360	CNS
CAV1	chr7	116227556	116229293	CNS
CAV1	chr7	116268838	116269440	CNS
CAV1	chr7	116273324	116274010	CNS
CAV1	chr7	116327276	116331188	CNS
CAV1	chr7	116331287	116334384	CNS
CAV1	chr7	116345275	116345781	CNS
CAV1	chr7	116345958	116349052	CNS
CAV1	chr7	116353124	116354955	CNS
CAV1	chr7	116355221	116357861	CNS
CAV1	chr7	116411044	116413659	CNS
CAV1	chr7	116415730	116419494	CNS
CAV1	chr7	116421735	116423410	CNS
CAV1	chr7	116341997	116343027	CNS
CAV1	chr7	116439854	116443746	CNS
CAV1	chr7	114429291	114431269	CNS
CAV1	chr7	114525449	114526553	CNS
CAV1	chr7	114529275	114530372	CNS
CAV1	chr7	114567780	114569045	CNS
CAV1	chr7	114569345	114571951	CNS
CAV1	chr7	114573849	114576684	CNS
CAV1	chr7	114583410	114585966	CNS
CAV1	chr7	114627975	114630075	CNS
CAV1	chr7	114648866	114650794	CNS
CAV1	chr7	114680511	114680894	CNS
CAV1	chr7	114870292	114872289	CNS
CAV1	chr7	114939946	114941012	CNS
CAV1	chr7	114992058	114993494	CNS
CAV1	chr7	115186013	115186673	CNS
CAV1	chr7	115301123	115304100	CNS
CAV1	chr7	115304366	115304820	CNS
CAV1	chr7	115310813	115313335	CNS
CAV1	chr7	115317812	115318384	CNS
CAV1	chr7	115318462	115319029	CNS
CAV1	chr7	115737105	115737568	CNS
CAV1	chr7	116502110	116504190	CNS

CAV1	chr7	116511658	116512938	CNS
CAV1	chr7	116551519	116552444	CNS
CAV1	chr7	116638294	116639676	CNS
CAV1	chr7	116645209	116646095	CNS
CAV1	chr7	116701473	116702466	CNS
CAV1	chr7	116738137	116738529	CNS
CAV1	chr7	116738883	116739229	CNS
CAV1	chr7	116764255	116764795	CNS
CAV1	chr7	116764919	116766070	CNS
CAV1	chr7	116771574	116774341	CNS
CAV1	chr7	116797868	116798377	CNS
CAV1	chr7	116869939	116871410	CNS
CAV1	chr7	116908705	116911053	CNS
CAV1	chr7	116923096	116924277	CNS
CAV1	chr7	117222645	117223108	CNS
CAV1	chr7	117305812	117306347	CNS
CAV1	chr7	117468242	117468792	CNS
CAV1	chr7	114458233	114460475	CNS
CAV1	chr7	114572709	114573331	CNS
CAV1	chr7	114785263	114786500	CNS
CAV1	chr7	115670883	115671400	CNS
CAV1	chr7	116899318	116900524	CNS
TBK1	chr12	64976982	64978649	Tissue-agnostic
TBK1	chr12	64849299	64852075	Tissue-agnostic
TBK1	chr12	64855048	64856317	Tissue-agnostic
TBK1	chr12	65194398	65194833	Tissue-agnostic
TBK1	chr12	65139887	65142519	Tissue-agnostic
TBK1	chr12	64349522	64350375	Tissue-agnostic
TBK1	chr12	64988381	64990575	Tissue-agnostic
TBK1	chr12	64852385	64854077	Tissue-agnostic
TBK1	chr12	65058182	65079989	Tissue-agnostic
TBK1	chr12	64479266	64484481	Tissue-agnostic
TBK1	chr12	64943421	64943781	Tissue-agnostic
TBK1	chr12	64953898	64955793	Tissue-agnostic
TBK1	chr12	64490881	64493995	Tissue-agnostic

Figure S1: qPCR measurement of TBK1 mRNA expression relative to mean expression in normal controls. Expression normalised relative to loading control. Related to Figure 1.

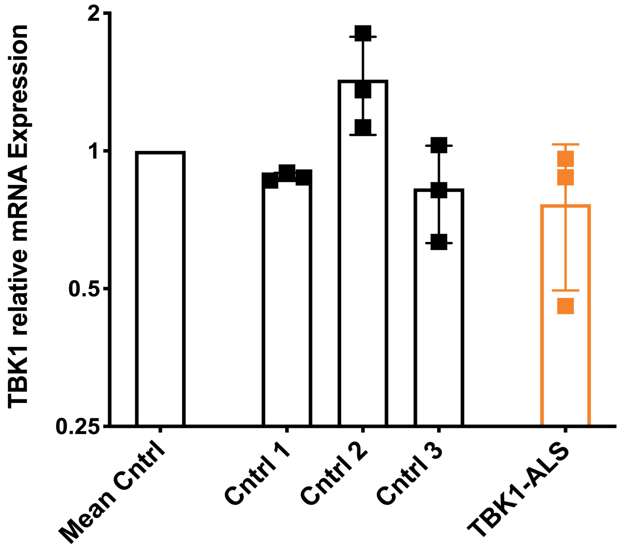

Table S2: Genetic variants wihin CAV1/CAV2 enhancer regions. D(i)=Discovery cohort; tissue-agnostic enhancers; D(ii)=Discovery cohort, CNS enhancers; R=Replicartion cohort.
Related to Figure 1.

Variants	ALS Frequency	Control Frequency	Cohort	Variants	ALS Frequency	Control Frequency	Cohort
chr7:115899988:T:C	0.00022	0	D(i)	chr7:115994719:G:A	0.000889878	0.001038961	D (ii)
chr7:115900605:A:G	0.00022	0	D(i)	chr7:115994770:C:G	0.000222469	0	D (ii)
chr7:115994269:C:T	0.00044	0	D(i)	chr7:115994869:C:T	0.000222469	0	D (ii)
chr7:115994303:G:A	0.00044	0	D(i)	chr7:115994875:C:T	0.000222469	0	D (ii)
chr7:115994310:A:G	0	0.00052	D(i)	chr7:115995110:A:G	0.000444939	0	D (ii)
chr7:115994595:T:G	0.00022	0	D(i)	chr7:115995386:G:A	0	0.000519481	D (ii)
chr7:115994627:T:C	0.00022	0	D(i)	chr7:115995411:C:G	0.000222469	0	D (ii)
chr7:115994719:G:A	0.00089	0.00104	D(i)	chr7:115996247:A:G	0.000222469	0	D (ii)
chr7:115994770:C:G	0.00022	0	D(i)	chr7:115996338:G:C	0.000222469	0	D (ii)
chr7:115994869:C:T	0.00022	0	D(i)	chr7:116035733:C:A	0.000444939	0	D (ii)
chr7:115994875:C:T	0.00022	0	D(i)	chr7:116152216:C:T	0.000222469	0.001038961	D (ii)
chr7:115995110:A:G	0.00044	0	D(i)	chr7:116175470:A:G	0.001557286	0.003116883	D (ii)
chr7:115995386:G:A	0	0.00052	D(i)	chr7:116175631:T:A	0	0.000519481	D (ii)
chr7:115995411:C:G	0.00022	0	D(i)	chr7:116175647:T:G	0.000222469	0	D (ii)
chr7:115996247:A:G	0.00022	0	D(i)	chr7:116181928:T:C	0	0.000519481	D (ii)
chr7:115996338:G:C	0.00022	0	D(i)	chr7:116182530:A:G	0.000222469	0	D (ii)
chr7:115996639:T:G	0.00022	0	D(i)	chr7:116182646:A:G	0.000667408	0.003116883	D (ii)
chr7:116034151:T:C	0.00022	0	D(i)	chr7:116182790:T:C	0	0.000519481	D (ii)
chr7:116035733:C:A	0.00044	0	D(i)	chr7:116211360:G:A	0.000444939	0.004675325	D (ii)
chr7:116152216:C:T	0.00022	0.00104	D(i)	chr7:116211574:C:T	0.000667408	0.000519481	D (ii)
chr7:116198357:C:T	0.00089	0	D(i)	chr7:116212628:A:G	0.000222469	0	D (ii)
chr7:116199522:T:C	0.00022	0	D(i)	chr7:116212861:A:G	0.000222469	0	D (ii)
chr7:116200589:T:A	0.00022	0	D(i)	chr7:116213876:A:T	0.000222469	0.001038961	D (ii)
chr7:116200705:C:T	0.00022	0	D(i)	chr7:116217283:C:G	0.000222469	0	D (ii)
chr7:116200719:C:A	0.00022	0	D(i)	chr7:116222625:T:C	0.000444939	0	D (ii)
chr7:116200953:A:T	0.00022	0	D(i)	chr7:116223328:A:G	0.000667408	0.000519481	D (ii)
chr7:116200959:G:T	0	0.00052	D(i)	chr7:116223448:C:T	0.000222469	0	D (ii)
chr7:116201160:T:C	0	0.00052	D(i)	chr7:116224152:G:A	0.002002225	0.007272727	D (ii)
chr7:116201160:T:G	0	0.00052	D(i)	chr7:116273953:C:T	0	0.000519481	D (ii)
chr7:116211360:G:A	0.00044	0.00468	D(i)	chr7:116273989:A:G	0.000222469	0.000519481	D (ii)
chr7:116211574:C:T	0.00067	0.00052	D(i)	chr7:116327730:T:C	0.000444939	0.000519481	D (ii)
chr7:116212628:A:G	0.00022	0	D(i)	chr7:116330812:T:C	0.000222469	0	D (ii)
chr7:116212861:A:G	0.00022	0	D(i)	chr7:116331129:T:C	0	0.000519481	D (ii)
chr7:116213876:A:T	0.00022	0.00104	D(i)	chr7:116333491:C:T	0.000222469	0	D (ii)
chr7:116217283:C:G	0.00022	0	D(i)	chr7:116333773:C:A	0	0.000519481	D (ii)
chr7:116222625:T:C	0.00044	0	D(i)	chr7:116342514:T:C	0.000222469	0	D (ii)
chr7:116223328:A:G	0.00067	0.00052	D(i)	chr7:116345315:A:G	0.000222469	0	D (ii)
chr7:116223448:C:T	0.00022	0	D(i)	chr7:116346600:T:C	0.000222469	0.000519481	D (ii)
chr7:116224152:G:A	0.00200	0.00727	D(i)	chr7:116347870:C:G	0.000222469	0	D (ii)
chr7:116474819:G:T	0.00067	0	D(i)	chr7:116355771:G:A	0.000222469	0	D (ii)
chr7:115957412:G:A	0.00022	0	D(i)	chr7:116356905:C:A	0.002892102	0.001038961	D (ii)
chr7:115957415:T:C	0.00022	0	D(i)	chr7:116356905:C:T	0.000222469	0.000519481	D (ii)
chr7:115957440:G:A	0.00022	0	D(i)	chr7:116357813:A:G	0.000444939	0	D (ii)

chr7:115957498:C:A	0.00022	0	D(i)	chr7:116412712:C:T	0.000222469	0	D (ii)
chr7:115957577:G:A	0.00022	0.00052	D(i)	chr7:116415873:G:T	0.000222469	0	D (ii)
chr7:116004321:G:A	0.00044	0	D(i)	chr7:116415881:T:G	0.000222469	0	D (ii)
chr7:116063605:A:G	0	0.00052	D(i)	chr7:116415938:C:G	0.000222469	0	D (ii)
chr7:116063624:T:C	0.00022	0	D(i)	chr7:116416799:C:T	0.001112347	0	D (ii)
chr7:116064199:T:C	0.00022	0	D(i)	chr7:116416941:A:G	0.000222469	0	D (ii)
chr7:116087637:G:A	0.00022	0	D(i)	chr7:116417019:A:G	0.000222469	0	D (ii)
chr7:116181928:T:C	0	0.00052	D(i)	chr7:116419236:G:T	0.001112347	0.001038961	D (ii)
chr7:116182530:A:G	0.00022	0	D(i)	chr7:116422352:C:T	0.000222469	0	D (ii)
chr7:116182646:A:G	0.00067	0.00312	D(i)	chr7:116422362:G:T	0.000222469	0	D (ii)
chr7:116182790:T:C	0	0.00052	D(i)	chr7:116422617:T:C	0.000889878	0.001558442	D (ii)
chr7:116184017:A:T	0.00912	0.00987	D(i)	chr7:116422623:C:A	0.005116796	0.002597403	D (ii)
chr7:116184130:T:A	0.00044	0	D(i)	chr7:116441514:T:C	0.000222469	0	D (ii)
chr7:116232009:G:C	0	0.00052	D(i)	chr7:116441539:C:T	0	0.000519481	D (ii)
chr7:116233640:T:C	0	0.00052	D(i)	chr7:116441559:C:A	0	0.000519481	D (ii)
chr7:114430787:C:G	0.008008899	0.008311688	D (ii)	chr7:116441604:C:A	0.003114572	0.001038961	D (ii)
chr7:114530323:G:A	0.000222469	0.000519481	D (ii)	chr7:116442636:T:A	0.000222469	0	D (ii)
chr7:114570572:C:A	0	0.000519481	D (ii)	chr7:116442680:C:T	0.000222469	0	D (ii)
chr7:114571016:G:A	0	0.000519481	D (ii)	chr7:116443482:C:T	0.000222469	0	D (ii)
chr7:114571375:G:C	0.000222469	0	D (ii)	chr7:116502532:G:C	0	0.001038961	D (ii)
chr7:114571427:G:T	0	0.000519481	D (ii)	chr7:116502617:C:G	0.000222469	0	D (ii)
chr7:114574678:G:A	0.000222469	0	D (ii)	chr7:116502985:G:C	0.000222469	0	D (ii)
chr7:114575111:A:C	0.001334816	0	D (ii)	chr7:116774049:T:C	0.000222469	0	D (ii)
chr7:114575748:A:G	0.000444939	0	D (ii)	chr7:116774169:C:T	0.000222469	0	D (ii)
chr7:114583529:T:C	0.000222469	0	D (ii)	chr7:116774262:G:T	0.004004449	0.000519481	D (ii)
chr7:114649995:C:A	0.000222469	0	D (ii)	chr7:116797872:G:A	0.000222469	0	D (ii)
chr7:114870400:T:G	0.000222469	0	D (ii)	chr7:116909821:G:A	0.000222469	0	D (ii)
chr7:114870987:C:T	0.000222469	0	D (ii)	chr7:116910136:C:A	0	0.000519481	D (ii)
chr7:114871000:G:C	0.000222469	0	D (ii)	chr7:116910162:C:G	0.000222469	0	D (ii)
chr7:114871004:A:T	0	0.000519481	D (ii)	chr7:116910505:A:C	0.000222469	0	D (ii)
chr7:114871090:C:T	0.000889878	0.001038961	D (ii)	chr7:117306108:T:C	0.000222469	0	D (ii)
chr7:114871798:T:C	0.000667408	0	D (ii)	chr7:117468457:C:T	0.000889878	0.001038961	D (ii)
chr7:114940467:A:G	0.000444939	0	D (ii)	chr7:117468773:A:G	0.000444939	0.000519481	D (ii)
chr7:114940537:T:C	0	0.000519481	D (ii)	chr7:114430749:G:T	0.000593472	0	R
chr7:114940606:C:T	0.000222469	0	D (ii)	chr7:114870400:T:G	0.001186944	0	R
chr7:115302267:G:A	0.000222469	0	D (ii)	chr7:114871090:C:T	0.000593472	0	R
chr7:115302388:C:T	0.000222469	0	D (ii)	chr7:114871798:T:C	0.000593472	0	R
chr7:115302422:A:G	0.000222469	0	D (ii)	chr7:114940077:A:G	0.000593472	0	R
chr7:115302425:G:T	0.000222469	0	D (ii)	chr7:114940422:T:A	0.000593472	0	R
chr7:115312834:C:T	0.000222469	0	D (ii)	chr7:115302422:A:G	0.000593472	0	R
chr7:115318030:G:A	0.000222469	0	D (ii)	chr7:115303637:A:G	0.000593472	0	R
chr7:115318066:G:A	0.001334816	0.000519481	D (ii)	chr7:115313234:T:G	0.000593472	0	R
chr7:115318198:G:A	0.000222469	0	D (ii)	chr7:115318066:G:A	0.002967359	0.002247191	R
chr7:115318232:C:T	0.000444939	0.000519481	D (ii)	chr7:115318495:C:T	0.000593472	0	R
chr7:115318257:A:T	0.000222469	0	D (ii)	chr7:115912442:G:A	0.000593472	0	R
chr7:115318267:C:G	0.000222469	0	D (ii)	chr7:115968420:C:T	0.001186944	0	R
chr7:115318323:G:C	0.000222469	0	D (ii)	chr7:115968431:A:G	0.000593472	0	R
chr7:115318339:G:A	0.000222469	0	D (ii)	chr7:115994330:C:T	0.000593472	0	R

chr7:115670928:A:G	0.001334816	0.001038961	D (ii)	chr7:115995441:A:C	0.000593472	0	R
chr7:115867898:G:A	0.000222469	0	D (ii)	chr7:116152067:C:T	0.000593472	0	R
chr7:115899988:T:C	0.000222469	0	D (ii)	chr7:116175470:A:G	0.000593472	0	R
chr7:115900605:A:G	0.000222469	0	D (ii)	chr7:116175798:A:G	0.000593472	0	R
chr7:115911421:A:G	0.000222469	0	D (ii)	chr7:116223751:C:G	0.001186944	0	R
chr7:115911791:T:G	0	0.000519481	D (ii)	chr7:116273806:G:A	0.000593472	0	R
chr7:115957412:G:A	0.000222469	0	D (ii)	chr7:116327730:T:C	0.000593472	0	R
chr7:115957415:T:C	0.000222469	0	D (ii)	chr7:116331111:T:C	0.000593472	0	R
chr7:115957440:G:A	0.000222469	0	D (ii)	chr7:116342272:T:C	0.000593472	0	R
chr7:115957498:C:A	0.000222469	0	D (ii)	chr7:116347329:T:C	0.000593472	0	R
chr7:115957577:G:A	0.000222469	0.000519481	D (ii)	chr7:116416799:C:T	0.000593472	0	R
chr7:115968420:C:T	0.000222469	0.000519481	D (ii)	chr7:116419236:G:T	0.001186944	0	R
chr7:115968429:G:A	0	0.000519481	D (ii)	chr7:116422617:T:C	0.000593472	0	R
chr7:115968452:C:T	0.000222469	0	D (ii)	chr7:116422623:C:A	0.007121662	0	R
chr7:115994269:C:T	0.000444939	0	D (ii)	chr7:116645747:A:C	0.000593472	0	R
chr7:115994303:G:A	0.000444939	0	D (ii)	chr7:116774262:G:T	0.005341246	0.004494382	R
chr7:115994310:A:G	0	0.000519481	D (ii)	chr7:116910136:C:A	0.000593472	0	R
chr7:115994595:T:G	0.000222469	0	D (ii)	chr7:117468457:C:T	0.000593472	0	R
chr7:115994627:T:C	0.000222469	0	D (ii)				

Figure S2: Increased dendrite length and altered PAX6 expression confirms successful neuronal differentiation of SH-SY5Y cells. Neuronal differentiated SH-SY5Y cells have increased dendrite length (A, *p<0.05, paired t-test, Forster et al., 2016). PAX6 is a transcription factor with an important role in neuronal development; reduced expression of PAX6 is concurrent with neuronal differentiation of Sh-SY5Y cells (B, Forster et al., 2016). Related to Figure 3**.**

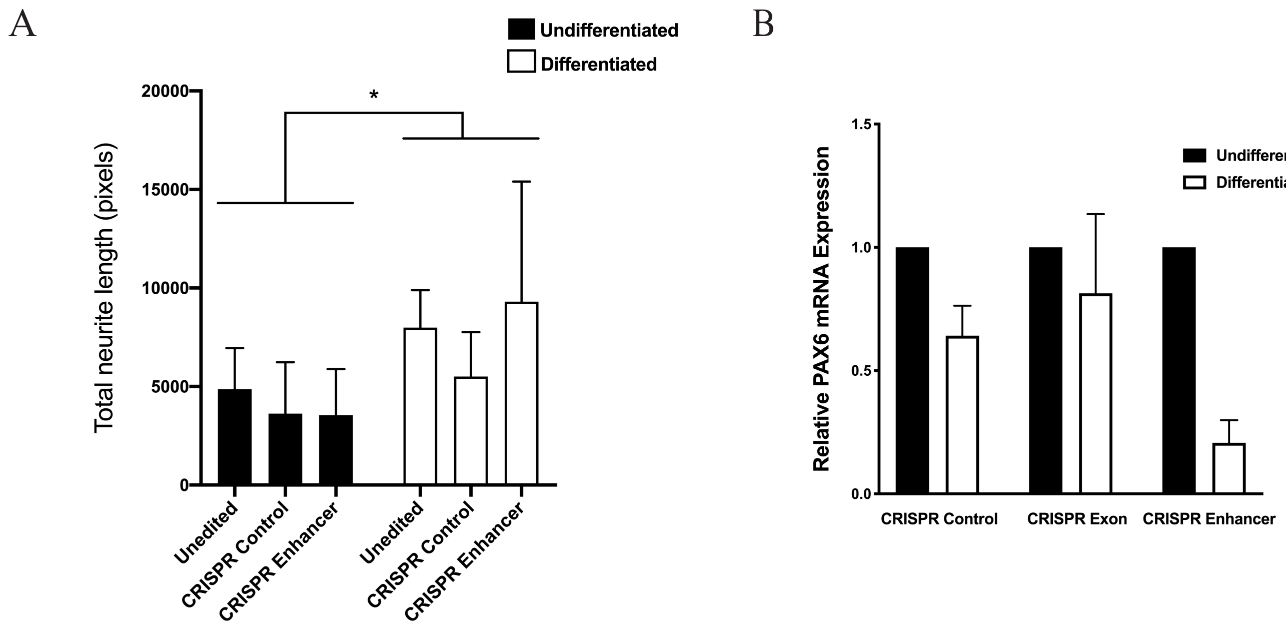

Table S3: DNA and RNA oligonucleotide sequences. Related to STAR Methods, 'CRISPR editing of mammalian cell lines'

**DNA oligos**	
qPCR RPL13A FWD	5'-CAAGCGGATGAACACCAACC-3'
qPCR RPL13A REV	5'-TTTTGTGGGGCAGCATACCT-3'
qPCR GAPDH FWD	5'-CAACTTTGGTATCGTGGAAGGAC-3'
qPCR GAPDH REV	5'-ACAGTCTTCTGGATGGCAGTG-3'
qPCR Pax6 FWD	5'-GTGCCCGTCCATCTTTGCTT-3'
qPCR Pax6 REV	5'-GCGCCCATCTGTTGGTTTTC-3'
qPCR CAV1 FWD	5’-CCAAGGAGATCGACCTGGTCAA– 3’
qPCR CAV1 REV	5’-GCCGTCAAAACTGTGTGTCCCT– 3’
qPCR CAV2	5'-TTCTCTTTGCCACCCTCAGCTG-3'
qPCR CAV2	5'-GAAGCATCGTCCTACGCTCGTA-3'
sequencing primers CAV enhancer 1 FWD	5'-ACCCTCCAGCACTAATGGACTT-3'
sequencing primers CAV enhancer 1 REV	5'-CCTGAGTTGATGACCCTTCTCCT-3'
sequencing primers CAV enhancer 2 FWD	5'-CTGCATACGCTATACCCGGC-3'
sequencing primers CAV enhancer 2 REV	5'-AGGTGTTTCGCTCCTCTGTC-3'
sequencing primers CAV exon FWD	5'-AGTACAGAGGGGTGTGGTGT-3'
sequencing primers CAV exon REV	5'-GGCTTACCTTGACCACGTCA-3'
**RNA Oligos (crRNA)**	
crRNA targeting CAV enhancer	5'-UUGUAAUCAGGAAUUUUCCA+modified linker-3'
crRNA targeting CAV exon	5'-AGUGUACGACGCGCACACCA+modified linker-3'

Table S4: Demographic information for patient-derived lymphoblastoid cell lines. Related to STAR Methods, 'Patient-derived lymphoblastoid cells'

	Genetic Variant	Age at collection (years)	Sex (M/F)
**CAV1-coding mutations**	p.Met111Val-*CAV1*	51	M
	p.Pro132Ala-*CAV1*	65	M
	p.Val155Ile-*CAV1*	50	F
**CAV1/CAV2-enhancer mutations**	chr7:116222625T>C	72	M
	chr7:115994269C>T	53	F
**TBK1-enhancer mutation**	chr12:65059913G>A	73	M
**ALS-controls**	-	78	M
	-	69	M
	-	49	F
**Neurologically normal controls**	-	48	F
	-	68	M
	-	56	M
