# Supplementary material for: Rare Variant Burden Analysis within Enhancers Identifies CAV1 as an ALS Risk Gene
Source: Cell Rep. 2020 Dec 1;33(9):108456. doi: 10.1016/j.celrep.2020.108456 (PMC7710676; doi:10.1016/j.celrep.2020.108456)
Supplement: Document S2. Article plus Supplemental Information [file mmc2.pdf]

Cell Reports

Report

Rare Variant Burden Analysis within Enhancers Identifies *CAV1* as an ALS Risk Gene

Graphical Abstract

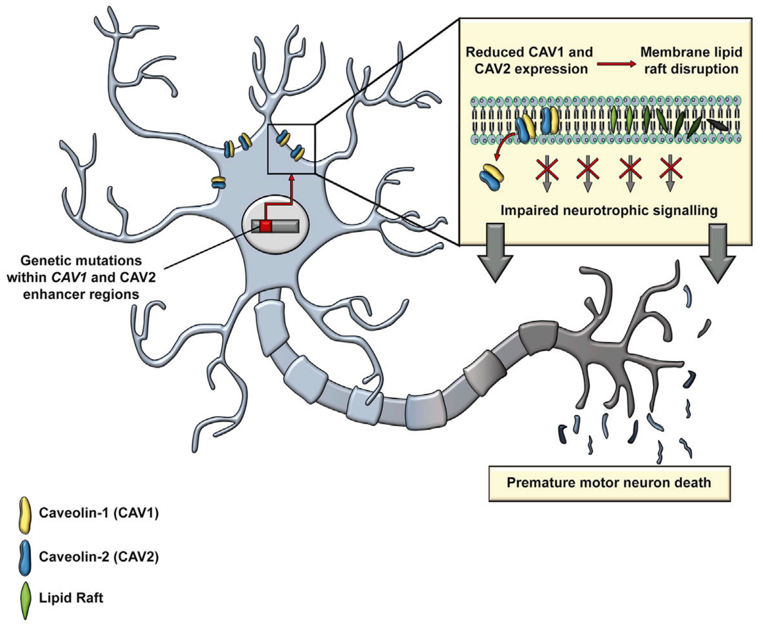

Highlights

- Identification of ALS-associated genetic variation within gene enhancers
- ALS-associated risk variants identified within enhancers and exons linked to *CAV1*
- *CAV1/CAV2* enhancer mutations reduce gene expression and disrupt membrane lipid rafts
- CRISPR-Cas9 perturbation of enhancer reduces *CAV1/CAV2* expression in neurons

Authors

Johnathan Cooper-Knock, Sai Zhang, Kevin P. Kenna, ..., Janine Kirby, Michael P. Snyder, Pamela J. Shaw

Correspondence

j.cooper-knock@sheffield.ac.uk (J.C.-K.),
pamela.shaw@sheffield.ac.uk (P.J.S.)

In Brief

Cooper-Knock et al. identify amyotrophic lateral sclerosis (ALS) risk variants within non-coding regulatory DNA linked to a known ALS gene, *TBK1*, but also *CAV1* and *CAV2*. Disease-associated variants reduce *CAV1/CAV2* expression and disrupt membrane lipid rafts with consequences for neurotrophic signaling. *CAV1* coding sequence also contains ALS-associated mutations.

Cooper-Knock et al., 2020, Cell Reports *33*, 108456
December 1, 2020 © 2020 The Author(s).
https://doi.org/10.1016/j.celrep.2020.108456

Cell Reports

CellPress OPEN ACCESS

Report

Rare Variant Burden Analysis within Enhancers Identifies *CAV1* as an ALS Risk Gene

Johnathan Cooper-Knock,[1,10,*] Sai Zhang,[2] Kevin P. Kenna,[3] Tobias Moll,[1] John P. Franklin,[1] Samantha Allen,[1] Helia Ghahremani Nezhad,[1] Alfredo Iacoangeli,[4] Nancy Y. Yacovzada,[5] Chen Eitan,[5] Eran Hornstein,[5] Eran Ehilak,[6] Petra Celadova,[7] Daniel Bose,[7] Sali Farhan,[8] Simon Fishilevich,[5] Doron Lancet,[5] Karen E. Morrison,[9] Christopher E. Shaw,[4] Ammar Al-Chalabi,[4] Project MinE ALS Sequencing Consortium, Jan H. Veldink,[3] Janine Kirby,[1] Michael P. Snyder,[2] and Pamela J. Shaw[1,*]

[1]Sheffield Institute for Translational Neuroscience (SITraN), University of Sheffield, Sheffield, UK
[2]Stanford Center for Genomics and Personalized Medicine, Department of Genetics, Stanford University School of Medicine, Stanford, CA 94305, USA
[3]Department of Neurology, Brain Center Rudolf Magnus, University Medical Center Utrecht, Utrecht, the Netherlands
[4]Department of Basic and Clinical Neuroscience, Institute of Psychiatry, Psychology and Neuroscience, King's College London, London, UK
[5]Department of Molecular Genetics, Weizmann Institute of Science, Rehovot, Israel
[6]Department of Biology, Lund University, Lund, Sweden
[7]Sheffield Institute for Nucleic Acids, University of Sheffield, Sheffield, UK
[8]Analytic and Translational Genetics Unit, Department of Medicine, Massachusetts General Hospital and Harvard Medical School, Boston, MA, USA
[9]Faculty of Medicine, University of Southampton, Southampton, UK
[10]Lead Contact
*Correspondence: j.cooper-knock@sheffield.ac.uk (J.C.-K.), pamela.shaw@sheffield.ac.uk (P.J.S.)
https://doi.org/10.1016/j.celrep.2020.108456

SUMMARY

Amyotrophic lateral sclerosis (ALS) is an incurable neurodegenerative disease. CAV1 and CAV2 organize membrane lipid rafts (MLRs) important for cell signaling and neuronal survival, and overexpression of CAV1 ameliorates ALS phenotypes *in vivo*. Genome-wide association studies localize a large proportion of ALS risk variants within the non-coding genome, but further characterization has been limited by lack of appropriate tools. By designing and applying a pipeline to identify pathogenic genetic variation within enhancer elements responsible for regulating gene expression, we identify disease-associated variation within *CAV1/CAV2* enhancers, which replicate in an independent cohort. Discovered enhancer mutations reduce *CAV1/CAV2* expression and disrupt MLRs in patient-derived cells, and CRISPR-Cas9 perturbation proximate to a patient mutation is sufficient to reduce *CAV1/CAV2* expression in neurons. Additional enrichment of ALS-associated mutations within *CAV1* exons positions *CAV1* as an ALS risk gene. We propose *CAV1/CAV2* overexpression as a personalized medicine target for ALS.

INTRODUCTION

Amyotrophic lateral sclerosis (ALS) is a universally fatal and relatively common neurodegenerative disease. Progress has been made in identification of highly penetrant coding-sequence mutations responsible for monogenic ALS, but the majority of sporadic ALS patients have no identified genetic risk factor despite heritability estimates as high as 52% (Ryan et al., 2019; Trabjerg et al., 2020). Importantly, discovery of genetic risk factors often leads to therapeutic targets.

ALS is defined by motor neuron death within the CNS; in motor neurons, caveolin 1 (CAV1) and caveolin 2 (CAV2) are expressed together in a hetero-oligomeric complex (de Almeida, 2017) within membrane lipid rafts (MLRs) on the cell surface and have a key role in organization of intercellular signaling (Sawada et al., 2019; Schmick and Bastiaens, 2014). CAV1 activity promotes neurotrophic signaling, leading to enhanced neuronal survival (Head et al., 2011; Mandyam et al., 2017). In contrast, loss of CAV1 accelerates neurodegeneration (Head et al., 2010, 2011). Abnormal neurotrophic signaling is well documented in ALS (Mutoh et al., 2000; Turner et al., 2004), and in particular, deficient neurotrophic signaling is associated with an increased vulnerability to neuronal injury (Bemelmans et al., 2006; Ghavami et al., 2014; Kowiański et al., 2018; Tooze and Schiavo, 2008). There are ongoing efforts to rebalance neurotrophic signaling in ALS patients (Berry et al., 2019); interestingly, neuronal overexpression of CAV1 improves survival and reduces motor neuron death in a mouse model of ALS (Sawada et al., 2019) and is being developed as a therapy for ALS (US patent no. 8969077B2) (Head et al., 2012).

Genome-wide association studies suggest a significant proportion of missing heritability for ALS is distributed throughout non-coding chromosomal regions (van Rheenen et al., 2016). Indeed, a large proportion of human DNA under evolutionary

This is an open access article under the CC BY license (http://creativecommons.org/licenses/by/4.0/).

constraint (Ward and Kellis, 2012) is non-coding, and mutations in non-coding DNA affect biological fitness (Graur, 2017), suggesting an important role in all aspects of cellular function. To date, genetic discoveries within the non-coding genome have been limited by a shortage of appropriate methodology.

The non-coding genome contains regions that regulate expression of coding genes; these regions include enhancers, which are *cis*-acting DNA sequences that modulate expression of target genes primarily through binding of transcription factors (TFs) (Koch et al., 2011). Physical interaction between an enhancer and the promoter of the target gene is mediated by DNA looping (Pennacchio et al., 2013). We have designed a pipeline for identification of disease-associated genetic variation within enhancers; variants are aggregated according to function, filtered based on evolutionary conservation (Hujoel et al., 2019), and collapsed into a single burden test (Cirulli and Goldstein, 2010).

We hypothesized that genetic variation within enhancers linked to expression of *CAV1* and *CAV2* would be associated with risk of ALS. Application of our pipeline confirmed our hypothesis and places this pathway upstream in the development of neurodegeneration.

RESULTS

Association of Regulatory Enhancer Elements with Coding Genes

Aggregation of genetic material with a common biological function improves power to detect genetic association via burden testing (Cirulli and Goldstein, 2010). We have aggregated sets of enhancers that regulate a common coding gene. As previously described (Fishilevich et al., 2017), we identified high-quality manually curated links between enhancers and coding genes based on agreement between correlated expression between genes, enhancer-RNAs (eRNAs), and TFs; expression quantitative trait loci (eQTL) within enhancers; capture Hi-C; and gene-enhancer genomic distances. Gene-enhancer relationships may be cell type specific (Heinz et al., 2015), whereas our method is cell and tissue agnostic. The disadvantage of this is that highly cell-specific relationships may be missed; however, by including data from multiple cell types, our method benefits from an increase in the quantity of high-quality training data. Moreover, many previously identified ALS-associated mutations are widely expressed (Cooper-Knock et al., 2013). Enhancers linked to *CAV1* and *CAV2* are detailed in Table S1.

A Pipeline for Testing for Disease-Associated Genetic Variation within Enhancers

Our pipeline for testing for disease-associated genetic variation within enhancers is detailed in Figure 1A. Following the aggregation of enhancers linked to individual coding genes, we filtered enhancer variants to remove those unlikely to be pathogenic prior to association testing. Enhancer variants were included if minor allele frequency (MAF) < 0.01 (Lek et al., 2016; van Rheenen et al., 2016) and LINSIGHT (Huang et al., 2017) score >0.8. LINSIGHT score >0.8 is consistent with strong evolutionary selection (Huang et al., 2017). Following filtering, case and control variant frequencies for each set of enhancers were collapsed into a single SKAT burden test (STAR Methods; Lee et al., 2012).

We tested our pipeline using whole-genome sequencing (WGS) data from 4,495 ALS cases and 1,925 controls (Project MinE; Data-Freeze-1). First, we hypothesized correctly that aggregated enhancers linked to all genes within the "amyotrophic lateral sclerosis" KEGG pathway (Kanehisa et al., 2017) would be enriched with ALS-associated genetic variation (SKAT-O; $p = 0.02$; 377 variants). Second, we hypothesized that pathogenic enhancer variants are likely to cause reduced transcription of their target coding gene, which might be expected to mimic a haploinsufficiency mechanism. Therefore, we examined variants within enhancers linked to expression of *TBK1*, which is uniquely known to cause ALS via haploinsufficiency (Freischmidt et al., 2015). Consistent with our hypothesis, genetic variation within *TBK1* enhancers is significantly associated with ALS ($p = 0.003$; SKAT-O; 12 variants; Table S1). Finally, expression of *TBK1* was reduced in patient-derived lymphoblastoid cells carrying an ALS-associated chr12:65059913G>A mutation within a TBK1 enhancer compared to mean expression in cells derived from neurologically normal controls, although this difference was not statistically significant (24% reduction; $p = 0.27$; Welch's t test; Figure S2).

Genetic Variation within *CAV1/CAV2* Enhancers Is Linked to ALS

We applied our pipeline to test for genetic association within enhancers linked to *CAV1* and *CAV2* expression. We discovered significant enrichment of ALS-associated genetic variation within enhancers linked to *CAV1* ($p = 3.88 \times 10^{-5}$; SKAT-O; 40 variants) and *CAV2* ($p = 1.52 \times 10^{-5}$; 57 variants). In total, 56 (1.2%) sporadic ALS patients carry one or more ALS-associated variants within *CAV1/CAV2* enhancers compared to 2 (0.1%) of controls (risk ratio = 12.0). There is significant overlap between enhancers and ALS-associated variants linked to *CAV1* and *CAV2* (Tables S1 and S3), which reflects shared function between the two proteins.

As a final test of our pipeline, we applied our analysis to all well-annotated genes found within KEGG pathways ($n = 3,761$). In this analysis, *CAV1* and *CAV2* enhancers carry the most significant enrichment with ALS-associated mutations compared to all other genes (Figure 1B). Importantly, there was no inflation of p values to indicate false positives (λGC 1,000 = 1.07).

Genetic Variation within CAV1 CNS Enhancers Is Associated with ALS

To test whether ALS-associated genetic variation within *CAV1/CAV2* enhancers is relevant within the CNS, we re-tested for genetic association using CNS-specific enhancers. A recent study released Hi-C data from CNS neurons (Rhie et al., 2018). We used these data to recalculate enhancer-gene relationships for *CAV1* (Table S1); no data were available for *CAV2*. Despite a significant change in the number and location of aggregated variants (Figure 1C), genetic variation within *CAV1* CNS enhancers was still significantly associated with ALS (SKAT-O; $p = 6.36\text{E}{-05}$; 128 variants; Table S3); 188 (4.1%) ALS patients

CellPress
OPEN ACCESS

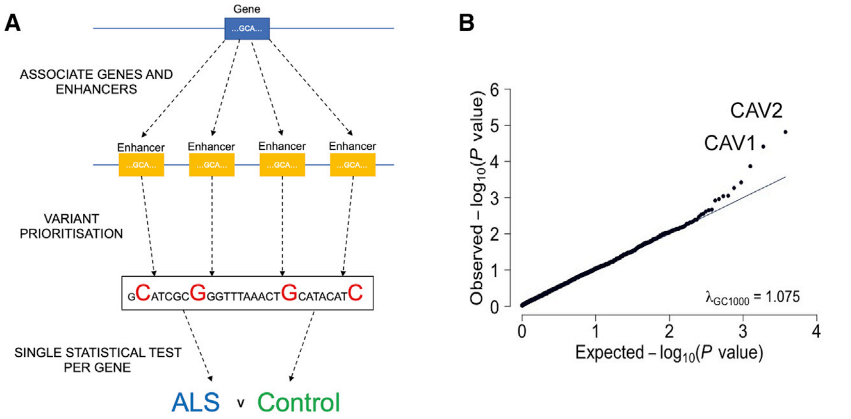

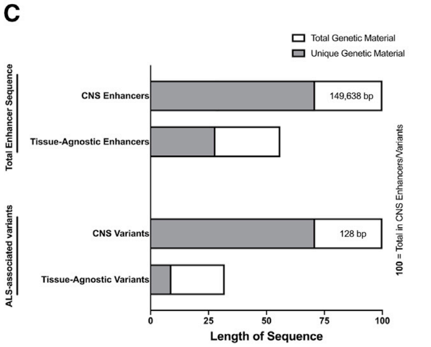

D

Sequence Change	ALS Frequency	Control Frequency
c.-182C>T	0.00022	0
p.Ile18Met	0.00022	0.00052
p.Lys30Gln	0.00022	0
p.Thr46Ala	0.00022	0
p.Gly77Arg	0.00022	0
p.Thr91Asn	0.00022	0
p.Ile109Thr	0.00022	0
p.Met111Val	0.00022	0
p.Pro132Ala	0.00022	0
p.Ile150Val	0.00022	0
p.Val155Ile	0.00089	0
p.Phe167Ser	0.00022	0

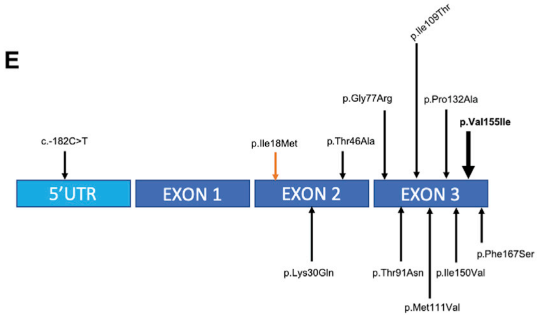

Figure 1. Significant Enrichment of ALS Genetic Risk within Enhancers and Coding Regions Linked to *CAV1* and *CAV2*

(A) Pipeline for variant filtering and burden testing; enhancers are first associated with genes based on epigenetic and transcriptome data (Fishilevich et al., 2017); enhancer variants are prioritized for further analysis if they are rare (MAF < 0.01; Lek et al., 2016) and evolutionary conserved (LINSIGHT score > 0.8; Huang et al., 2017).

(B) Q-Q plot depicting on x axis the −log 10 of expected p value versus the actually measured p value for 3,761 enhancer groups using whole-genome sequencing (WGS) data from 4,495 ALS cases and 1,925 controls. *CAV1/CAV2* enhancers deviate from the null distribution (diagonal), revealing that the burden of variants measured in *CAV1/CAV2* enhancers is significantly associated with risk of ALS even after correction for multiple testing.

(C) Quantity of genetic material (bp) relative to CNS enhancers derived from Hi-C data (Rhie et al., 2018); CNS enhancers = 100. Upper two bars denote total genetic material; lower two bars denote ALS-associated genetic variants only. Gray shading denotes material unique to CNS or tissue-agnostic enhancers versus material shared by both (white).

(D and E) *CAV1*-coding variants passing filtering criteria are depicted in the table and figure. This analysis utilized WGS data from 4,495 ALS cases and 1,925 controls. One variant is present at higher frequency in controls (orange arrow), and one variant is present is multiple ALS patients (bold arrow); all other variants were discovered in a single ALS patient and zero controls.

carried an ALS-associated *CAV1* CNS enhancer risk variant compared to 17 (0.9%) of controls (risk ratio = 4.6).

Replication of ALS-Associated Genetic Variation within CAV1 and CAV2 Enhancers

To validate observed genetic association within *CAV1/CAV2* enhancers, we obtained WGS data from an independent cohort of 1,685 ALS patients and 445 controls (Project MinE; Data-Freeze-2). Derived tissue-agnostic *CAV1/CAV2* enhancers were not sufficiently variable in this smaller cohort (<10 variants), though Hi-C-derived CNS enhancers contained more genetic material. Re-applying our pipeline to *CAV1* Hi-C-derived CNS enhancers in the validation cohort revealed significant enrichment of ALS-associated genetic variation ($p = 0.03$; SKAT-O; 51 variants; Table S3); 60 (3.6%) ALS patients carried an ALS-associated *CAV1* CNS enhancer risk variant compared to 3 (0.7%) of controls (risk ratio = 5.1).

As a secondary analysis, we obtained summary statistics from 32,298 European non-Finnish non-ALS controls (Karczewski et al., 2020; Taliun et al., 2019). A larger sample size facilitated re-analysis of tissue-agnostic *CAV1/CAV2* enhancers within the same ALS cohort but using an independent population matched control cohort. Re-applying our pipeline revealed significant enrichment of ALS-associated genetic variants within *CAV1* and *CAV2* enhancers compared to the discovery cohort (n = 4,495, Data-Freeze-1; *CAV1*: $p = 2.64 \times 10^{-9}$, SKAT-O, 112 variants; *CAV2*: $p = 7.30 \times 10^{-8}$, SKAT-O, 174 variants) and replication cohort (n = 1,685, Data-Freeze-2; *CAV1*: $p = 8.6 \times 10^{-5}$, SKAT-O, 94 variants; *CAV2*: $p = 4.87 \times 10^{-7}$, SKAT-O, 150 variants).

Genetic Variation within *CAV1* Coding Sequence Is Associated with ALS

It is likely that genetic variation within linked enhancer and coding regions can produce similar phenotypes. We tested for ALS-associated genetic variation within *CAV1* and *CAV2* exons by rare-variant burden testing using WGS data from 4,495 ALS cases and 1,925 controls (Project MinE; Data-Freeze-1). In addition to filtering by MAF < 0.01 (van Rheenen et al., 2016), we

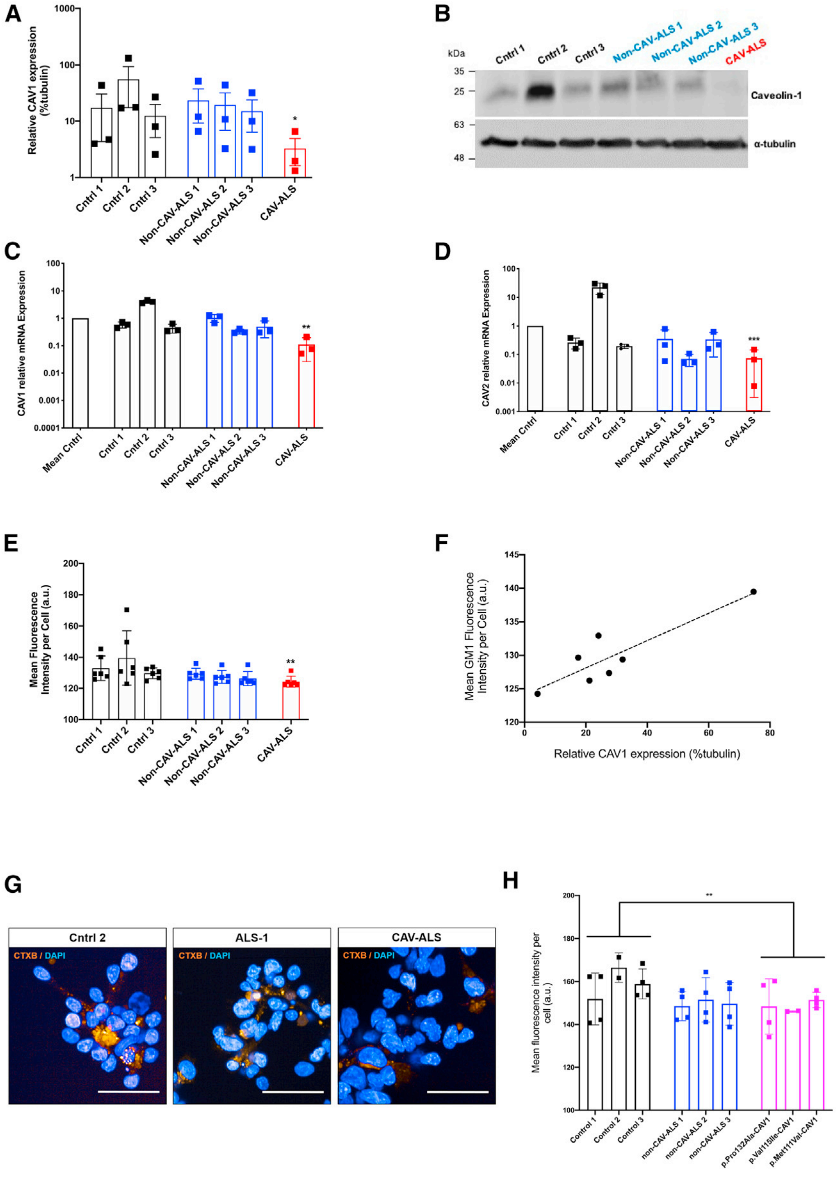

Figure 2. Patient-Derived Lymphoblastoid Cell Lines Carrying an ALS-Associated *CAV*-Enhancer/Coding Variants Have Reduced Expression of *CAV1*/*CAV2* and Disrupted MLR

Lymphoblastoid cells were derived from neurologically normal controls (n = 3, black), ALS patients without *CAV*-enhancer variants (n = 3, blue), ALS patients carrying *CAV1*-coding mutations (n = 3, magenta), and cells carrying a disease-associated chr7:116222625T>C-*CAV1*/*CAV2* enhancer variant (red).

(A and B) Immunoblotting measurement of CAV1 protein expression relative to α-tubulin loading control with an example blot.

(C and D) qPCR measurement of *CAV1* and *CAV2* mRNA expression relative to mean expression in normal controls; expression normalized relative to loading control.

(E, G, and H) Measurement of MLR integrity by live-cell imaging and GM1 labeling with CTxB. CTxB fluorescence is plotted with example images. Scale bar, 50 μm.

(F) CAV1 protein expression is plotted versus MLR integrity as measured by CTxB fluorescence in the same cell line, with regression line (dotted).

Data presented as mean ± 1 SD. $^{*}p < 0.05$; $^{**}p < 0.01$; $^{***}p < 0.001$.

introduced a functional filter to identify variants that alter protein function (STAR Methods; Cingolani et al., 2012). In *CAV1*, but not *CAV2*, coding sequence, we identified significant enrichment of functional genetic variation in ALS patients (p = 0.03; 12 variants; Firth logistic regression; beta = 1.47; Figures 1D and 1E). In total, 15 (0.3%) ALS patients carried a *CAV1* coding variant compared to 1 (0.05%) of controls (risk ratio = 6.4). Coding and enhancer mutations occurred in independent individuals, which excludes the possibility that the observed convergence is a consequence of linkage disequilibrium.

Reduced *CAV1* and *CAV2* Expression in Patient-Derived Cells Carrying an ALS-Associated Enhancer Variant

Burden testing derives power from aggregating mutations into a single statistical test, but as a consequence, experimental evaluation is necessary to determine which individual mutations are pathogenic: indeed, it is likely that a significant proportion of variants are not pathogenic (Lee et al., 2012). Reduced CAV1 expression is toxic to neurons (Head et al., 2010, 2011), and therefore, we measured *CAV1*/*CAV2* expression in lymphoblastoid cells derived from ALS patients carrying *CAV1*/*CAV2* enhancer variants: chr7:116222625T>C and chr7:115994269:C>T (Table S3).

In cells carrying chr7:116222625T>C, CAV1 protein (89% reduction; p = 0.05; Mann-Whitney test; Figures 2A and 2B) and mRNA (89% reduction; p = 0.003; Welch's t test; Figure 2C) and *CAV2* mRNA (93% reduction; p = 0.002; Welch's t test; Figure 2D) expression was significantly reduced compared to mean expression in cells derived from neurologically normal controls. Unfortunately, immunoblotting for CAV2 was not possible due to lack of a sufficiently specific antibody. Expression was also reduced compared to ALS patients without an enhancer mutation (Figures 2A–2D).

Cells carrying chr7:115994269:C > T did not show reduced expression of *CAV1*/*CAV2* (data not shown). We speculate that this mutation may impact transcription only in CNS cells, or alternatively, this variant may be non-functional.

Impaired MLR Formation in Patient-Derived Cells Carrying an ALS-Associated Enhancer Variant

Reduced CAV1/CAV2 function is proposed to be toxic via disruption of MLR, leading to impaired cell signaling

CellPress
OPEN ACCESS

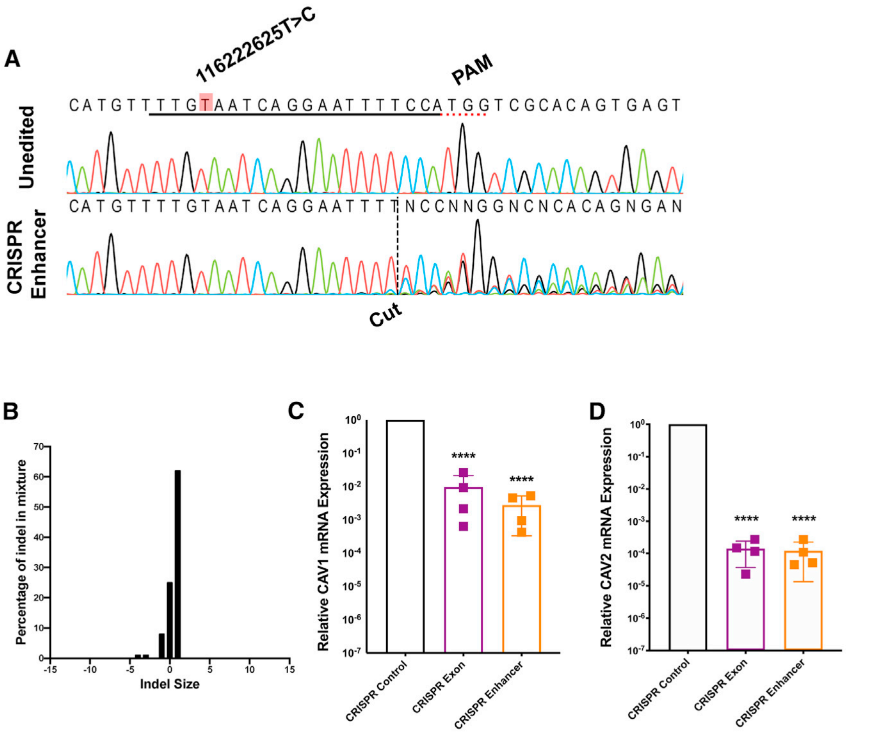

Figure 3. CRISPR-Directed Perturbation of a *CAV*-Enhancer Region Proximate to a Patient Mutation Reduces *CAV1/CAV2* Expression in a Differentiated SH-SY5Y Neuronal Cell

(A) Sanger sequencing traces demonstrating spCas9 cut site adjacent to PAM and subsequent waveform decomposition in enhancer edited cells. Position of chr7:116222625T>C mutation is indicated. Black line indicates gRNA sequence.

(B) Indel distribution within *CAV*-enhancer region in CRISPR-edited SH-SY5Y cells.

(C and D) qPCR measurement of *CAV1* mRNA and *CAV2* mRNA reveals reduced expression in *CAV*-enhancer and *CAV1*-exon CRISPR-edited neurons compared to CRISPR editing of *HPRT*; expression normalized relative to loading control.

Data presented as mean ± 1 SD. ****$p < 0.0001$.

(Sawada et al., 2019). We tested whether ALS-associated enhancer variants that reduce *CAV1/CAV2* expression also impair MLR formation. MLR integrity was measured by expression of GM1 gangliosides as labeled by cholera-toxin B (CTxB) (Aman et al., 2001; Day and Kenworthy, 2015; Sawada et al., 2019). CTxB fluorescence is significantly reduced in lymphoblastoid cells carrying chr7:116222625T>C compared to cells derived from neurologically normal controls (8% reduction; Figure 2E); fluorescence was also reduced compared to ALS patients without a *CAV1/CAV2* enhancer variant (Figures 2E and 2G). Strikingly, in all cell lines, GM1 expression and CAV1 protein expression are positively correlated ($r = 0.89$; $p = 0.007$; Pearson correlation; Figure 2F), which is consistent with direct dependence of MLR integrity on CAV1 function.

We hypothesized that *CAV1*-coding variants would produce a similar effect on MLR formation. Consistent with this, CTxB fluorescence is significantly reduced in lymphoblastoid cells carrying p.Met111Val-, p.Pro132Ala-, and p.Val155Ile-CAV1 mutations compared to cells derived from neurologically normal controls ($p = 0.009$; t test; Figure 2H).

CRISPR-SpCas9 Enhancer Editing Reduces *CAV1/CAV2* Expression in Neurons

We have confirmed that patient-derived cells carrying an ALS-associated *CAV1/CAV2* enhancer mutation display reduced *CAV1/CAV2* expression and disrupted MLR, which is likely to lead to neurotoxicity (Sawada et al., 2019). However, these experiments were carried out in non-neuronal cells. To confirm that disruption of the same enhancer is sufficient to reduce *CAV1/CAV2* expression in a human CNS-relevant neuronal cell, we used CRISPR-SpCas9 editing to introduce indel mutations proximal to the site of the chr7:116222625T>C mutation in SH-SY5Y cells, which were subsequently differentiated into neurons.

Guide RNAs (gRNAs) were designed to target a protospacer adjacent motif (PAM) site 16 bp downstream of the chr7:116222625T>C mutation site. Sanger sequencing and waveform decomposition analysis (Hsiau et al., 2018) revealed 72% editing efficiency in undifferentiated SH-SY5Y cells (Figure 3A). The majority of introduced changes were a single-nucleotide insertion (chr7:116222638T>TT; Figure 3B). A commercially available gRNA targeting *CAV1* exon 2 was chosen to introduce a nonsense mutation and served as a positive control, and a commercially available control gRNA targeting *HPRT* served as a negative control. CRISPR-SpCas9-edited SH-SY5Y cells were differentiated to a neuronal phenotype; successful differentiation was confirmed by altered expression of PAX6 (Figure S4A; Forster et al., 2016) and increased total dendritic length ($p = 0.01$; paired t test; Figure S4B; Forster et al., 2016). Differentiated cells were harvested, and RNA was extracted for qPCR. We confirmed reduced expression of *CAV1* (>99% reduction; $p < 0.0001$; Welch's t test) and *CAV2* (>99% reduction; $p < 0.0001$) mRNA in enhancer edited cells (Figures 3C and 3D). *CAV2* mRNA expression was reduced in the context of enhancer editing but also by *CAV1*-coding editing, which likely reflects interdependence between the two genes (Drab et al., 2001). Extreme reductions in *CAV1/CAV2* expression are notable; however, phenotypic change in excess of editing efficiency is well described and may be a consequence of CRISPR interference (Gaj et al., 2017).

ALS-Associated *CAV1/CAV2* Enhancer Mutation Is Associated with TF Binding Sites

Pathogenic enhancer mutations may act via altered TF binding (Karnuta and Scacheri, 2018). To identify potential changes in TF binding associated with *CAV1/CAV2* enhancer mutations, we analyzed publicly available ChIP sequencing data (https://genome.ucsc.edu; TF ChIP-seq Clusters from ENCODE 3, version: 3 November 2018). We identified five DNA-binding

proteins associated with both the site of the chr7:116222625T>C mutation and the cut site for our CRISPR-SpCas9-editing experiment in at least one cell type: RAD21; CTCF; FOS; SMC3; and CEBPB. However, introduced mutations did not significantly alter the predicted strength of TF binding (STAR Methods; Nguyen et al., 2018).

DISCUSSION

Genetic discoveries in ALS have focused on high effect variants within coding genes in patients with autosomal dominant inheritance. The non-coding genome is thought to contain missing heritability (Pallares, 2019). We developed an approach to discover genetic association within gene enhancer elements. Using our methodology, we successfully identified and validated ALS-associated genetic variation within enhancer and coding regions associated with *CAV1*; we therefore propose *CAV1* to be an ALS risk gene. It is notable that we did not identify ALS-associated nonsense mutations within *CAV1* or *CAV2*. *CAV1/CAV2* nonsense mutations would dramatically reduce gene expression irrespective of cell type or even developmental stage and may manifest a broad range of phenotypes, including motor neuron toxicity, but not specifically ALS. A traditional focus only on loss-of-function coding variants may therefore have missed the link between *CAV1/CAV2* expression and ALS.

Our work builds upon previous observations that CAV1 function is neuroprotective in neurodegenerative disease (Head et al., 2010) and in ALS in particular (Sawada et al., 2019). Until now, it was not clear whether CAV1 dysfunction was a cause or effect of neuronal toxicity; our discovery of genetic risk associated with *CAV1/CAV2* expression places this pathway upstream in the development of disease. Using patient-derived cells, we have shown that ALS-associated genetic variation within *CAV1/CAV2* enhancers and *CAV1* coding sequence reduces *CAV1/CAV2* expression and disrupts MLR, which is contingent with impaired neurotrophic signaling and consequently neurodegeneration (Sawada et al., 2019). Moreover, CRISPR-SpCas9 perturbation proximate to a *CAV/CAV2* enhancer mutation reduced *CAV1* and *CAV2* expression in human neuronal cells, suggesting that this enhancer region is functional within the CNS.

Enhancer function is thought to depend on the binding of TFs (Koch et al., 2011). Current understanding of function within enhancer regions is limited (Levo et al., 2015), in part because of a paucity of variants with validated biological impact. This incomplete understanding is reflected in our failure to link a change in TF binding to the changes in *CAV1/CAV2* expression we observe. Our discovery forms a platform for improved understanding of molecular function within these regions. We propose that our approach, namely the discovery of disease-associated genetic variation, is a means of overcoming a reliance on unphysiological *in vitro* assays to understand enhancer biology (Gasperini et al., 2020).

Our genetic study was performed in sporadic ALS cases. As a result, we propose that the mutations we have identified are likely to be risk factors rather than fully penetrant monogenic causes of disease. Indeed, sporadic ALS is proposed to be a multistep process involving both genetic and environmental insults (Al-Chalabi et al., 2014). The association of CAV1 function with neurotrophic signaling is consistent with this premise; identified deficient neurotrophic signaling in ALS has been proposed as a risk factor that increases the vulnerability of motor neurons to additional insults (Bemelmans et al., 2006; Ghavami et al., 2014; Kowiański et al., 2018; Tooze and Schiavo, 2008).

The premise of personalized medicine for complex disease is that gene-environment interactions leading to disease are likely to be heterogeneous (Li et al., 2019). We suggest that, in a significant number of ALS patients, genetic mutations leading to reduced CAV1/CAV2 function are a significant cause of neuronal toxicity. Upregulation of CAV1 is in development as a therapeutic tool (Head et al., 2011; US patent no. 8969077B2); our data suggest that this could be applied to genetically selected ALS patients in a personalized medicine approach.

STAR★METHODS

Detailed methods are provided in the online version of this paper and include the following:

- KEY RESOURCES TABLE
- RESOURCE AVAILABILITY
  - Lead Contact
  - Materials Availability
  - Data and Code Availability
- EXPERIMENTAL MODEL AND SUBJECT DETAILS
  - Selection of patients and controls for genetic sequencing
  - SH-SY5Y neuroblastoma cells
  - Patient-derived lymphoblastoid cells
- METHOD DETAILS
  - High Throughput DNA sequencing and QC
  - Variant Filtering
  - Cell lysis
  - Immunoblotting
  - CRISPR editing of mammalian cell lines
  - Determining CRISPR editing efficiency
  - Quantitative PCR (RT-PCR):
  - SH-SY5Y neuronal differentiation
  - Immunocytochemistry
  - Live cell imaging
- QUANTIFICATION AND STATISTICAL ANALYSIS
  - Immunoblotting and quantitative PCR
  - Burden testing
  - Modeling of TF binding

SUPPLEMENTAL INFORMATION

Supplemental Information can be found online at https://doi.org/10.1016/j.celrep.2020.108456.

CONSORTIA

Project MinE ALS Sequencing consortium includes Ian Blair, Naomi Wray, Matthew Kiernan, Miguel Mitne Neto, Adriano Chio, Ruben Cauchi, Wim Robberecht, Philip van Damme, Phillipe Corcia, Phillipe Couratier, Orla Hardiman, Russel McLaughlin, Marc Gotkine, Vivan Drory, Nicola Ticozzi, Vincenzo Silani, Jan Veldink, Leonard van den Berg, Mamede de Carvalho, Jesus Mora Pardina, Monica Povedano, Peter Andersen, Markus Wber, Nazli Başak, Ammar Al-Chalabi, Christopher Shaw, Pamela Shaw, Karen Morrison, John Landers, and Jonathan Glass.

ACKNOWLEDGMENTS

This project has received funding from the European Research Council (ERC) under the European Union's Horizon 2020 research and innovation program (no. 772376 - EScORIAL). The project is supported through the following funding organizations under the aegis of JPND: Medical Research Council (MR/L501529/1 and MR/R024804/1) and Economic and Social Research Council (ES/L008238/1). The collaboration project is co-funded by the PPP Allowance made available by Health~Holland, Top Sector Life Sciences & Health to stimulate public-private partnerships. We acknowledge support from a Lee Newton PhD studentship (T.M.), "My Name"5 Doddie Foundation (J.P.F.), the Wellcome Trust (J.C.-K., 216596/Z/19/Z; D.B., 213501/Z/18/Z), and NIHR (P.J.S. and A.A.C.). This work was also supported by the NIHR Sheffield Biomedical Research Centre for Translational Neuroscience. Biosample collection was supported by the MND Association and the Wellcome Trust (P.J.S.). We are very grateful to those ALS patients and control subjects who generously donated biosamples.

AUTHOR CONTRIBUTIONS

J.C.-K., S.Z., K.P.K., N.Y.Y., C.E., D.B., E.H., J.K., M.P.S., and P.J.S. were responsible for the conception and design of the study. J.C.-K., T.M., J.P.F., S. Farhan, H.G.N., S.A., K.E.M., A.A.-C., C.E.S., D.B., P.C., S. Fishilevich, D.L., K.PK., J.H.V., J.K., and P.J.S. were responsible for data acquisition. J.C.-K., T.M., J.P.F., S. Fishilevich, S.Z., A.I., K.P.K., H.G.N., S.A., N.Y.Y., C.E., E.H., S.F., and D.L. were responsible for analysis of data. J.C.-K., S.Z., K.P.K., T.M., A.I., J.P.F., S. Farhan, S.A., N.Y.Y., C.E., E.H., S. Fishilevich, D.L., J.H.V., J.K., M.P.S., and P.J.S. were responsible for interpretation of data. The Project MinE ALS Sequencing consortium was involved in data acquisition and analysis. All authors meet the four ICMJE authorship criteria and were responsible for revising the manuscript, approving the final version for publication, and for accuracy and integrity of the work.

DECLARATION OF INTERESTS

The authors declare no competing interests.

Received: May 13, 2020
Revised: September 15, 2020
Accepted: November 9, 2020
Published: December 1, 2020

REFERENCES

Al-Chalabi, A., Calvo, A., Chio, A., Colville, S., Ellis, C.M., Hardiman, O., Heverin, M., Howard, R.S., Huisman, M.H.B., Keren, N., et al. (2014). Analysis of amyotrophic lateral sclerosis as a multistep process: a population-based modelling study. Lancet Neurol. *13*, 1108–1113.

Aman, A.T., Fraser, S., Merritt, E.A., Rodigherio, C., Kenny, M., Ahn, M., Hol, W.G.J., Williams, N.A., Lencer, W.I., and Hirst, T.R. (2001). A mutant cholera toxin B subunit that binds GM1- ganglioside but lacks immunomodulatory or toxic activity. Proc. Natl. Acad. Sci. USA *98*, 8536–8541.

Arganda-Carreras, I., Fernández-González, R., Muñoz-Barrutia, A., and Ortiz-De-Solorzano, C. (2010). 3D reconstruction of histological sections: Application to mammary gland tissue. Microsc. Res. Tech. *73*, 1019–1029.

Basu, S., and Pan, W. (2011). Comparison of statistical tests for disease association with rare variants. Genet. Epidemiol. *35*, 606–619.

Bemelmans, A.-P., Husson, I., Jaquet, M., Mallet, J., Kosofsky, B.E., and Gressens, P. (2006). Lentiviral-mediated gene transfer of brain-derived neurotrophic factor is neuroprotective in a mouse model of neonatal excitotoxic challenge. J. Neurosci Res. *83*, 50–60.

Berry, J.D., Cudkowicz, M.E., Windebank, A.J., Staff, N.P., Owegi, M., Nicholson, K., McKenna-Yasek, D., Levy, Y.S., Abramov, N., Kaspi, H., et al. (2019). NurOwn, phase 2, randomized, clinical trial in patients with ALS: Safety, clinical, and biomarker results. Neurology *93*, e2294–e2305.

Brooks, B.R. (1994). El Escorial World Federation of Neurology criteria for the diagnosis of amyotrophic lateral sclerosis. Subcommittee on Motor Neuron Diseases/Amyotrophic Lateral Sclerosis of the World Federation of Neurology Research Group on Neuromuscular Diseases and the El Escorial "Clinical limits of amyotrophic lateral sclerosis" workshop contributors. J. Neurol. Sci. *124* (*Suppl*), 96–107.

Chang, C.C., Chow, C.C., Tellier, L.C., Vattikuti, S., Purcell, S.M., and Lee, J.J. (2015). Second-generation PLINK: rising to the challenge of larger and richer datasets. Gigascience *4*, 7.

Cingolani, P., Platts, A., Wang, L.L., Coon, M., Nguyen, T., Wang, L., Land, S.J., Lu, X., and Ruden, D.M. (2012). A program for annotating and predicting the effects of single nucleotide polymorphisms, SnpEff: SNPs in the genome of Drosophila melanogaster strain w1118; iso-2; iso-3. Fly (Austin) *6*, 80–92.

Cirulli, E.T., and Goldstein, D.B. (2010). Uncovering the roles of rare variants in common disease through whole-genome sequencing. Nat. Rev. Genet. *11*, 415–425.

Cooper-Knock, J., Jenkins, T., and Shaw, P.J. (2013). Clinical and Molecular Aspects of Motor Neuron Disease (Biota Publishing).

Danecek, P., Auton, A., Abecasis, G., Albers, C.A., Banks, E., DePristo, M.A., Handsaker, R.E., Lunter, G., Marth, G.T., Sherry, S.T., et al.; 1000 Genomes Project Analysis Group (2011). The variant call format and VCFtools. Bioinformatics *27*, 2156–2158.

Day, C.A., and Kenworthy, A.K. (2015). Functions of cholera toxin B-subunit as a raft cross-linker. Essays Biochem. *57*, 135–145.

de Almeida, C.J.G. (2017). Caveolin-1 and Caveolin-2 can be antagonistic partners in inflammation and beyond. Front. Immunol. *8*, 1530.

Drab, M., Verkade, P., Elger, M., Kasper, M., Lohn, M., Lauterbach, B., Menne, J., Lindschau, C., Mende, F., Luft, F.C., et al. (2001). Loss of caveolae, vascular dysfunction, and pulmonary defects in caveolin-1 gene-disrupted mice. Science *293*, 2449–2452.

Fishilevich, S., Nudel, R., Rappaport, N., Hadar, R., Plaschkes, I., Iny Stein, T., Rosen, N., Kohn, A., Twik, M., Safran, M., et al. (2017). GeneHancer: genome-wide integration of enhancers and target genes in GeneCards. Database (Oxford) *2017*, bax028.

Forster, J.I., Köglsberger, S., Trefois, C., Boyd, O., Baumuratov, A.S., Buck, L., Balling, R., and Antony, P.M.A. (2016). Characterization of Differentiated SH-SY5Y as Neuronal Screening Model Reveals Increased Oxidative Vulnerability. J. Biomol Screen. *21*, 496–509.

Freischmidt, A., Wieland, T., Richter, B., Ruf, W., Schaeffer, V., Müller, K., Marroquin, N., Nordin, F., Hübers, A., Weydt, P., et al. (2015). Haploinsufficiency of TBK1 causes familial ALS and fronto-temporal dementia. Nat. Neurosci. *18*, 631–636.

Gaj, T., Ojala, D.S., Ekman, F.K., Byrne, L.C., Limsirichai, P., and Schaffer, D.V. (2017). In vivo genome editing improves motor function and extends survival in a mouse model of ALS. Sci. Adv. *3*, eaar3952.

Gasperini, M., Tome, J.M., and Shendure, J. (2020). Towards a comprehensive catalogue of validated and target-linked human enhancers. Nat. Rev. Genet. *21*, 292–310.

Ghavami, S., Shojaei, S., Yeganeh, B., Ande, S.R., Jangamreddy, J.R., Mehrpour, M., Christoffersson, J., Chaabane, W., Moghadam, A.R., Kashani, H.H., et al. (2014). Autophagy and apoptosis dysfunction in neurodegenerative disorders. Prog. Neurobiol. *112*, 24–49.

Graur, D. (2017). An upper limit on the functional fraction of the human genome. Genome Biol. Evol. *9*, 1880–1885.

Head, B.P., Peart, J.N., Panneerselvam, M., Yokoyama, T., Pearn, M.L., Niesman, I.R., Bonds, J.A., Schilling, J.M., Miyanohara, A., Headrick, J., et al. (2010). Loss of caveolin-1 accelerates neurodegeneration and aging. PLoS ONE *5*, e15697.

Head, B.P., Hu, Y., Finley, J.C., Saldana, M.D., Bonds, J.A., Miyanohara, A., Niesman, I.R., Ali, S.S., Murray, F., Insel, P.A., et al. (2011). Neuron-targeted caveolin-1 protein enhances signaling and promotes arborization of primary neurons. J. Biol. Chem. *286*, 33310–33321.

Head, B.P., Patel, P.M., Patel, H., and Roth, M. (2012). Neuronal specific targeting of caveolin expression to restore synaptic signaling and improve cognitive function in the neurodegenerative brain and motor function in spinal cord. US patent 8969077B2, filed November 7, 2011, and granted May 10, 2012.

Heinz, S., Romanoski, C.E., Benner, C., and Glass, C.K. (2015). The selection and function of cell type-specific enhancers. Nat. Rev. Mol. Cell Biol. *16*, 144–154.

Hoerndli, F.J., Toigo, M., Schild, A., Götz, J., and Day, P.J. (2004). Reference genes identified in SH-SY5Y cells using custom-made gene arrays with validation by quantitative polymerase chain reaction. Anal. Biochem. *335*, 30–41.

Hruz, T., Wyss, M., Docquier, M., Pfaffl, M.W., Masanetz, S., Borghi, L., Verbrugghe, P., Kalaydjieva, L., Bleuler, S., Laule, O., et al. (2011). RefGenes: identification of reliable and condition specific reference genes for RT-qPCR data normalization. BMC Genomics *12*, 156.

Hsiau, T., Conant, D., Rossi, N., Maures, T., Waite, K., Yang, J., Joshi, S., Kelso, R., Holden, K., Enzmann, B.L., et al. (2018). Inference of CRISPR edits from Sanger trace data. bioRxiv. https://doi.org/10.1101/251082.

Huang, Y.-F., Gulko, B., and Siepel, A. (2017). Fast, scalable prediction of deleterious noncoding variants from functional and population genomic data. Nat. Genet. *49*, 618–624.

Hujoel, M.L.A., Gazal, S., Hormozdiari, F., van de Geijn, B., and Price, A.L. (2019). Disease heritability enrichment of regulatory elements is concentrated in elements with ancient sequence age and conserved function across species. Am. J. Hum. Genet. *104*, 611–624.

Kanehisa, M., Furumichi, M., Tanabe, M., Sato, Y., and Morishima, K. (2017). KEGG: new perspectives on genomes, pathways, diseases and drugs. Nucleic Acids Res. *45* (*D1*), D353–D361.

Karczewski, K.J., Francioli, L.C., Tiao, G., Cummings, B.B., Alföldi, J., Wang, Q., Collins, R.L., Laricchia, K.M., Ganna, A., Birnbaum, D.P., et al. (2020). The mutational constraint spectrum quantified from variation in 141,456 humans. Nature *581*, 434–443.

Karnuta, J.M., and Scacheri, P.C. (2018). Enhancers: bridging the gap between gene control and human disease. Hum. Mol. Genet. *27* (*R2*), R219–R227.

Koch, F., Fenouil, R., Gut, M., Cauchy, P., Albert, T.K., Zacarias-Cabeza, J., Spicuglia, S., de la Chapelle, A.L., Heidemann, M., Hintermair, C., et al. (2011). Transcription initiation platforms and GTF recruitment at tissue-specific enhancers and promoters. Nat. Struct. Mol. Biol. *18*, 956–963.

Kowiański, P., Lietzau, G., Czuba, E., Waśkow, M., Steliga, A., and Moryś, J. (2018). BDNF: A Key Factor with Multipotent Impact on Brain Signaling and Synaptic Plasticity. Cell Mol. Neurobiol. *38*, 579–593.

Lee, S., Emond, M.J., Bamshad, M.J., Barnes, K.C., Rieder, M.J., Nickerson, D.A., Christiani, D.C., Wurfel, M.M., and Lin, X.; NHLBI GO Exome Sequencing Project—ESP Lung Project Team (2012). Optimal unified approach for rare-variant association testing with application to small-sample case-control whole-exome sequencing studies. Am. J. Hum. Genet. *91*, 224–237.

Lek, M., Karczewski, K.J., Minikel, E.V., Samocha, K.E., Banks, E., Fennell, T., O'Donnell-Luria, A.H., Ware, J.S., Hill, A.J., Cummings, B.B., et al.; Exome Aggregation Consortium (2016). Analysis of protein-coding genetic variation in 60,706 humans. Nature *536*, 285–291.

Levo, M., Zalckvar, E., Sharon, E., Dantas Machado, A.C., Kalma, Y., Lotam-Pompan, M., Weinberger, A., Yakhini, Z., Rohs, R., and Segal, E. (2015). Unraveling determinants of transcription factor binding outside the core binding site. Genome Res. *25*, 1018–1029.

Li, J., Li, X., Zhang, S., and Snyder, M. (2019). Gene-environment interaction in the era of precision medicine. Cell *177*, 38–44.

Longair, M.H., Baker, D.A., and Armstrong, J.D. (2011). Simple Neurite Tracer: open source software for reconstruction, visualization and analysis of neuronal processes. Bioinformatics *27*, 2453–2454.

Mandyam, C.D., Schilling, J.M., Cui, W., Egawa, J., Niesman, I.R., Kellerhals, S.E., Staples, M.C., Busija, A.R., Risbrough, V.B., Posadas, E., et al. (2017). Neuron-targeted caveolin-1 improves molecular signaling, plasticity, and behavior dependent on the hippocampus in adult and aged mice. Biol. Psychiatry *81*, 101–110.

McCall, M.N., McMurray, H.R., Land, H., and Almudevar, A. (2014). On non-detects in qPCR data. Bioinformatics *30*, 2310–2316.

Mutoh, T., Sobue, G., Hamano, T., Kuriyama, M., Hirayama, M., Yamamoto, M., and Mitsuma, T. (2000). Decreased phosphorylation levels of TrkB neurotrophin receptor in the spinal cords from patients with amyotrophic lateral sclerosis. Neurochem Res. *25*, 239–245.

Nguyen, N.T.T., Contreras-Moreira, B., Castro-Mondragon, J.A., Santana-Garcia, W., Ossio, R., Robles-Espinoza, C.D>, Bahin, M., Collombet, S., Vincens, P., Thieffry, D., et al. (2018). RSAT 2018: regulatory sequence analysis tools 20th anniversary. Nucleic. Acids Res. *46*, W209–W214.

Pallares, L.F. (2019). Searching for solutions to the missing heritability problem. eLife *8*, e53018.

Pennacchio, L.A., Bickmore, W., Dean, A., Nobrega, M.A., and Bejerano, G. (2013). Enhancers: five essential questions. Nat. Rev. Genet. *14*, 288–295.

Project MinE ALS Sequencing Consortium (2018). Project MinE: study design and pilot analyses of a large-scale whole-genome sequencing study in amyotrophic lateral sclerosis. Eur. J. Hum. Genet. *26*, 1537–1546.

Purcell, S., Neale, B., Todd-Brown, K., Thomas, L., Ferreira, M.A.R., Bender, D., Maller, J., Sklar, P., de Bakker, P.I.W., Daly, M.J., and Sham, P.C. (2007). PLINK: a tool set for whole-genome association and population-based linkage analyses. Am. J. Hum. Genet. *81*, 559–575.

Raczy, C., Petrovski, R., Saunders, C.T., Chorny, I., Kruglyak, S., Margulies, E.H., Chuang, H.-Y., Källberg, M., Kumar, S.A., Liao, A., et al. (2013). Isaac: ultra-fast whole-genome secondary analysis on Illumina sequencing platforms. Bioinformatics *29*, 2041–2043.

Rhie, S.K., Schreiner, S., Witt, H., Armoskus, C., Lay, F.D., Camarena, A., Spitsyna, V.N., Guo, Y., Berman, B.P., Evgrafov, O.V., et al. (2018). Using 3D epigenomic maps of primary olfactory neuronal cells from living individuals to understand gene regulation. Sci. Adv. *4*, eaav8550.

Ryan, M., Heverin, M., McLaughlin, R.L., and Hardiman, O. (2019). Lifetime risk and heritability of amyotrophic lateral sclerosis. JAMA Neurol. *76*, 1367–1374.

Sawada, A., Wang, S., Jian, M., Leem, J., Wackerbarth, J., Egawa, J., Schilling, J.M., Platoshyn, O., Zemljic-Harpf, A., Roth, D.M., et al. (2019). Neuron-targeted caveolin-1 improves neuromuscular function and extends survival in $SOD1^{G93A}$ mice. FASEB J. *33*, 7545–7554.

Schmick, M., and Bastiaens, P.I.H. (2014). The interdependence of membrane shape and cellular signal processing. Cell *156*, 1132–1138.

Schmittgen, T.D., and Livak, K.J. (2008). Analyzing real-time PCR data by the comparative C(T) method. Nat. Protoc. *3*, 1101–1108.

Taliun, D., Harris, D.N., Kessler, M.D., Carlson, J., Szpiech, Z.A., Torres, R., Taliun, S.A.G., Corvelo, A., Gogarten, S.M., Kang, H.M., et al. (2019). Sequencing of 53,831 diverse genomes from the NHLBI TOPMed Program. bioRxiv. Published online March 6, 2019. https://doi.org/10.1101/563866.

Tooze, S.A., and Schiavo, G. (2008). Liaisons dangereuses: autophagy, neuronal survival and neurodegeneration. Curr. Opin. Neurobiol. *18*, 504–515.

Trabjerg, B.B., Garton, F.C., van Rheenen, W., Fang, F., Henderson, R.D., Mortensen, P.B., Agerbo, E., and Wray, N.R. (2020). ALS in Danish registries: heritability and links to psychiatric and cardiovascular disorders. Neurol. Genet. *6*, e398.

Turner, B.J., Murray, S.S., Piccenna, L.G., Lopes, E.C., Kilpatrick, T.J., and Cheema, S.S. (2004). Effect of p75 neurotrophin receptor antagonist on disease progression in transgenic amyotrophic lateral sclerosis mice. J. Neurosci. Res. *78*, 193–199.

van Rheenen, W., Shatunov, A., Dekker, A.M., McLaughlin, R.L., Diekstra, F.P., Pulit, S.L., van der Spek, R.A., Võsa, U., de Jong, S., Robinson, M.R., et al.; PARALS Registry; SLALOM Group; SLAP Registry; FALS Sequencing Consortium; SLAGEN Consortium; NNIPPS Study Group (2016). Genome-wide association analyses identify new risk variants and the genetic architecture of amyotrophic lateral sclerosis. Nat. Genet. *48*, 1043–1048.

Ward, L.D., and Kellis, M. (2012). Evidence of abundant purifying selection in humans for recently acquired regulatory functions. Science *337*, 1675–1678.

CellPress
OPEN ACCESS

STAR★METHODS

KEY RESOURCES TABLE

Reagent or Resource	Source	Identifier
Antibodies		
Anti-Caveolin-1	GeneTex	#GTX100205; RRID:AB_1240559
Anti-Caveolin-1	Abcam	#AB2910; RRID:AB_303405
α-tubulin	Sigma	#T9026; RRID:AB_477593
Anti-Pax6	Abcam	#AB5790; RRID:AB_305110
Anti-mouse HRP-conjugate	Promega	#W4021; RRID:AB_430834
Anti-rabbit HRP-conjugate	Promega	#W4011; RRID:AB_430833
Donkey anti-rabbit Alexa568 secondary	Invitrogen	#A10042; RRID:AB_2534017
Donkey anti-mouse Alexa488 secondary	Invitrogen	#A-21202; RRID:AB_141607
Chemicals, Peptides and Recombinant Proteins		
Alt-R® S.p. Cas9 Nuclease V3	Integrated DNA technologies	#1081059
Alt-R® Cas9 Electroporation Enhancer	Integrated DNA technologies	#1075915
Dulbecco's Modified Eagle medium	Lonza	#12-604F
RPMI-1640 medium with L-glutamine	Lonza	#BE12-702F
Neurobasal medium	ThermoFisher Scientific	#12348017
Penicillin-Streptomycin	Sigma	#P4333
Fibronectin	Merck	#FC010
10x Trypsin	Sigma	#59427C
Foetal bovine serum	ThermoFisher Scientific	#10270106
L-glutamine (200mM)	ThermoFisher Scientific	#25030081
Agarose	Melford	#MB1200
Ethidium bromide solution	Sigma	#E1510
VeriFi mix red	PCRBio	#PB10.42-01
Tri reagent	Sigma	#93289-100ML
M-MLV reverse transcriptase	ThermoFisher Scientific	#28025-013
5x First Strand buffer	ThermoFisher Scientific	#18057-018
0.1M Dithiothreitol	ThermoFisher Scientific	#707265ML
dNTP Mix	ThermoFisher Scientific	#10534823
SYBR Green Brilliant III master mix	Agilent	#600882
Random hexamer primer	ThermoFisher Scientific	#SO142
Pre-stained protein ladder	Abcam	#116028
Bradford reagent	Bio-Rad	#5000001
Laemmli buffer	Bio-Rad	#1610747
b-mercaptoethanol	Sigma	#M6250
EDTA	Sigma	#E5134
HEPES	Sigma	#H3375
SigmaFAST Protease Inhibitor Cocktail tablets	Sigma	#S8820
PMSF protease inhibitor	ThermoFisher Scientific	#36978
Clarity Western ECL blotting substrate	Bio-Rad	#1705060S
TracrRNA	Integrated DNA technologies	#1072533
TE Buffer, RNase-free pH 8	ThermoFisher Scientific	#AM9849
Dulbecco's Phosphate Buffered Saline	Sigma	#D8537-500ML
Triton X-100	Sigma-Aldrich	#T8787
Normal horse serum	Vector	#S-2000-20
Hoechst 33342	ThermoFisher Scientific	#62249

(*Continued on next page*)

Continued		
Reagent or Resource	Source	Identifier
All-trans retinoic acid	Sigma	#R2625
BDNF	PeproTech	#450-02
N-2 supplement	ThermoFisher Scientific	#17502048
Cholera Toxin Subunit B (recombinant) Alexa555 Conjugate	Invitrogen	#C22843
Critical Commercial Assays		
Pierce BCA Assay Protein Assay Kit	ThermoFisher Scientific	#23225
GenElute Mammalian Genomic DNA Miniprep Kit	Sigma	#G1N350
Direct-zol RNA Miniprep Kit	Zymo Research	#R2050
Neon Transfection System 10 μL Kit	ThermoFisher Scientific	#MPK1096
Alt-R CRISPR-Cas9 Control Kit, Human, 2 nmol	Integrated DNA technologies	#1072554
Experimental Models: Cell Lines		
SH-SY5Y	ATCC	Cat.#CRL-2266
Patient and control LCL lines	MNDA (UK) DNA Bank	N/A
Oligonucleotides		
See Supplementary Table S5		N/A
Software and Algorithms		
SKAT	https://cran.r-project.org/web/packages/SKAT/index.html	N/A
R	https://cran.r-project.org/mirrors.html	N/A
Galaxy	https://usegalaxy.org/	N/A
snpStats	https://www.bioconductor.org/packages/release/bioc/html/snpStats.html	N/A
VariantAnnotation	https://www.bioconductor.org/packages/release/bioc/html/VariantAnnotation.html	N/A
Plink	http://zzz.bwh.harvard.edu/plink/download.shtml	N/A
PRISM 7	GraphPad	N/A
ICE CRISPR analysis tool	https://ice.synthego.com/#/	N/A
CRISPOR guide RNA design tool	http://crispor.tefor.net/	N/A
CFX Maestro	Bio-Rad	N/A
Harmony High-Content Imaging and Analysis Software	PerkinElmer	N/A
FIJI (FIJI Is Just ImageJ)	NIH	N/A

RESOURCE AVAILABILITY

Lead Contact

Further information and requests for resources and reagents should be directed to and will be fulfilled by the corresponding author, Johnathan Cooper-Knock (j.cooper-knock@sheffield.ac.uk).

Materials Availability

All unique/stable reagents generated in this study are available from the Lead Contact without restriction.

Data and Code Availability

This study did not generate any unique datasets or code. Whole genome sequencing data is available through Project MinE (https://www.projectmine.com/research/data-sharing/). A data access committee controls access to raw data, ensuring a FAIR data setup (https://www.datafairport.org).

EXPERIMENTAL MODEL AND SUBJECT DETAILS

Selection of patients and controls for genetic sequencing

All 6,180 patients and 2,370 controls included in this study were recruited at specialized neuromuscular centers in the UK, Belgium, Germany, Ireland, Italy, Spain, Turkey, the United States and the Netherlands (Project MinE ALS Sequencing Consortium, 2018).

Patients were diagnosed with possible, probable or definite ALS according to the 1994 El-Escorial criteria (Brooks, 1994). All controls were free of neuromuscular diseases and matched for age, sex and geographical location. Analysis focused on *Data-Freeze-1* including 4,495 ALS patients and 1,925 controls; *Data-Freeze-2* (released December 2019) was used for validation. After excluding population outliers *Data-Freeze-2* included 1,685 ALS patients and 445 controls.

Secondary analysis was performed using summary statistics derived from WGS of 32,298 European non-Finnish controls. This cohort and the relevant analysis pipeline have been previously described (Karczewski et al., 2020).

The study was approved by the South Sheffield Research Ethics Committee. Also, this study followed study protocols approved by Medical Ethical Committees for each of the participating institutions. Written informed consent was obtained from all participating individuals. All methods were performed in accordance with relevant national and international guidelines and regulations.

SH-SY5Y neuroblastoma cells

Human SH-SY5Y neuroblastoma cells were cultured in Dulbecco's Modified Eagle's Medium (DMEM) (Lonza) supplemented with 10% (v/v) fetal bovine serum (FBS) (Thermo-Fisher Scientific), 50 units/mL of penicillin and 50 μg/mL of streptomycin. Cell lines were maintained at 5% CO2 in a 37°C incubator and split every 3-4 days. All experimental work was performed using cells within the range of 7-32 passages.

Patient-derived lymphoblastoid cells

Lymphoblastoid cell lines derived from Caucasian ALS patients (n = 9) and neurologically normal controls (n = 3), all of Northern European descent, were obtained from the UK Motor Neurone Disease Association (MNDA) DNA Bank. Demographic details are provided in Table S6. Lymphoblastoid cells were cultured in RPMI 1640 Medium (Lonza) supplemented with 2mM L-glutamine and 10% (v/v) FBS. Cells were maintained at 5% CO_2 in a 37°C incubator and split every 3-4 days.

METHOD DETAILS

High Throughput DNA sequencing and QC

Methods are described elsewhere (Project MinE ALS Sequencing Consortium, 2018). In brief, DNA was extracted from venous blood samples and quality was assessed by gel electrophoresis. DNA samples were sequenced using Illumina's FastTrack services (San Diego, CA, USA) on the Illumina HiSeq 2000 platform. Sequencing was 100 bp paired-end (~40 × coverage) performed using PCR-free library preparation. The Isaac pipeline (Raczy et al., 2013) was used for alignment to the hg19 reference genome as well as to call single nucleotide variants (SNVs), insertions and deletions (indels), and larger structural variants (SVs). Variants not passing Isaac's quality filter were set to missing; non-autosomal chromosome and multi-allelic variants were excluded. Sample and SNP QC were performed using PLINK (Chang et al., 2015; Purcell et al., 2007) and VCFtools (Danecek et al., 2011). Samples were excluded if missingness by sample < 10% across all 22 chromosomes. Remaining sample QC steps were performed on a set of high-quality biallelic SNPs that had minor allele frequency (MAF) > 10%, missingness < 0.1%, were LD-pruned at an r^2 threshold of 0.2, were not A/T or C/G SNPs, did not lie in the major histocompatibility complex (MHC) or LCT locus, and did not occur in the inversions on chromosome 8 or chromosome 17. The ~30,000 SNPs overlapping this set of SNPs and HapMap 3 (HM3) were used to calculate principal components projecting the ALS cases and controls onto the HM3 samples. Samples of non-European ancestry, defined as further than 10 standard deviations from the European-ancestry populations in HM3, were excluded from further analysis. Samples with an inbreeding coefficient > 3 s.d. from the mean of the distribution were excluded, as were unexpected related samples. Samples with discordant sex information (comparing chromosome X genotypes and phenotype information) were excluded.

Variants with missingness > 5% were removed, as were variants out of Hardy-Weinberg equilibrium in controls ($p < 1 \times 10^{-6}$) and monomorphic variants (induced by sample exclusions). Differential missingness between cases and controls was checked and variants with $p < 1 \times 10^{-6}$ were removed. Variants with extreme depth of coverage (> 6 s.d. from the mean of the total depth distribution) were also excluded. Finally, the mitochondrial, X and Y chromosomes were excluded from analysis. Approximately 10 million sites were lost during variant QC.

Variant Filtering

ALS features a polygenic rare variant architecture (van Rheenen et al., 2016); therefore all searches for pathogenic variants in enhancer and coding regions featured a filter for MAF within the Genome Aggregation Database (gnomAD) of < 1/100 control alleles (Lek et al., 2016). Additional filtering varied between area reflecting differences in function. In enhancer regions variants were included only if evolutionary conserved based on a LINSIGHT score > 0.8 (Huang et al., 2017). In coding regions we filtered for variants with impact on protein function as defined by snpeff (Cingolani et al., 2012): Variants annotated HIGH/MODERATE/LOW impact were included, but we excluded variants annotated 'synonymous' or 'TF_binding_site_variant' because these functions are independent of amino acid sequence.

Cell lysis

Lymphoblastoid cells were lysed in urea lysis buffer [8M urea; 1% (w/v) DTT; 20% (w/v) SDS; 1.5M Tris pH 6.8; + dH_2O) + PIC (20μL/mL) + 1mM PMSF at room temperature (RT). Samples were sonicated at 50% amplitude for 10 s (SoniPrep 150, MSE) followed

by a 30 s incubation at RT (3x). Samples were then incubated at RT for 15 minutes. Lysates were centrifuged at 17,000xg for 5 minutes at RT. Total protein concentration within the supernatant was quantified using a Pierce BCA Protein Assay Kit (ThermoFisher Scientific) according to the manufacturer's instructions and absorbance was measured at 562nm on a PHERAstar FS spectrophotometer (BMG Biotech). Lysates were mixed with 4x Laemmli buffer (277.8mM Tris-HCl; 44.4% (v/v) glycerol; 4.4% SDS; 0.02% bromophenol blue; 355mM 2-mercaptoethanol; pH 6.8) and boiled at 95°C for 5 minutes. Protein extracts were fractionated on 12% SDS polyacrylamide gels and electrophoretically transferred to nitrocellulose membranes.

Immunoblotting

Nitrocellulose membranes were initially blocked in 5% (w/v) milk (Marvel)/Tris Buffered Saline, with Tween® 20 (TBST) (20mM Tris, 137mM NaCl, 0.2% (v/v) Tween® 20, pH 7.6) for 1 hour at RT then probed using the relevant primary antibody overnight at 4°C. Anti-caveolin 1 (GeneTex) (1:500 dilution) and α-tubulin (Abcam) (1:2000 dilution) primary antibodies were detected using a horseradish peroxidase (HRP)-conjugated rabbit secondary antibody and a HRP-conjugated mouse secondary antibody, respectively (Promega) (1:5000 dilution). Protein bands were visualized using ECL substrate (Bio-Rad) and the chemiluminescence signal was imaged using a G:BOX (Syngene).

CRISPR editing of mammalian cell lines

Guide RNAs (gRNAs) were designed using the Crispor tool (http://crispor.tefor.net/) to target CAV-enhancer regions. Design was guided by proximity to patient enhancer mutation sites, available protospacer adjacent motifs (PAM), and predicted on- and off-target efficiencies. gRNAs targeting within 30bp either side of the patient enhancer mutation site (chr7:116222625, hg19) were considered and screened for editing efficiency as below. One guide sequence (5′ -UUGUAAUCAGGAAUUUUCCA-3′) was most efficient and chosen for subsequent experimentation. Validated, commercially available CRISPR control targeting HPRT (IDT) and CAV1 exon-targeting (ThermoFisher Scientific) gRNAs were also obtained (Table S5). gRNA duplexes were assembled from tracrRNA and crRNA in a thermocycler according to manufacturer's instructions under RNase-free conditions. Cells were cultured to ensure 70%–90% confluency on the day of transfection. 1ml antibiotic-free DMEM (Lonza) was prepared and incubated in 24-well plates at 37°C. CRISPR/Cas9 Ribonucleoproteins were formed by complexing 240ng gRNA duplex with 1250ng Alt-R V3 Cas9 Protein (IDT) in 10 μL buffer R (from 10 μL Neon transfection kit, ThermoFisher Scientific) - a 1:1 molar ratio - for 10 minutes. 100,000 viable cells were aliquoted per transfection and centrifuged at 400 x g for 4 minutes. Cells were washed in calcium- and magnesium-free Dulbecco's Phosphate Buffered Saline (Sigma) and centrifuged at 400 x g for 4 minutes. Cell pellets were resuspended in 10 μL buffer R containing Cas9 protein and gRNA duplexes. 2 μL of 10.8 μM electroporation enhancer (IDT) was added and the solution mixed thoroughly to ensure a suspension of single cells. 10 μL of this mixture was loaded into a Neon transfection system (ThermoFisher Scientific) and electroporated according to manufacturer's instructions (1200V, 3 pulse, 20 s pulse width for SH-SY5Y cells). Cells were then transferred to pre-warmed media in 24-well plates.

Determining CRISPR editing efficiency

Genomic DNA was isolated from CRISPR-edited and control cells using a GenElute Mammalian DNA Miniprep Kit (Sigma) according to manufacturer's instructions. A ~400bp region around the expected cas9 cut site was amplified by polymerase chain reaction using VeriFi mix (PCRbio). Expected amplification was confirmed using gel electrophoresis, and the products were Sanger-sequenced. Sequencing trace files were uploaded to ICE (https://ice.synthego.com) and an indel efficiency calculated.

Quantitative PCR (RT-PCR):

Cells were cultured until at least 70% confluent, lysed on ice using an appropriate volume of Tri Reagent (Sigma) for 5 minutes and transferred to 1.5ml RNase-free tubes. Total RNA was extracted using a Direct-zol RNA Miniprep Kit (Zymo) according to manufacturer's instructions, and RNA concentration confirmed using a NanoDrop spectrophotometer (ThermoFisher Scientific). 2 μg of total RNA was then converted to cDNA by adding 1 μL 10mM dNTPs, 1 μL 40 μM random hexamer primer (ThermoFisher Scientific), and DNase/RNase-free water to a total volume of 14 μL. This mixture was heated for 5 minutes at 70°C then placed on ice for 5 minutes. 4 μL of 5x FS buffer, 2 μL 0.1M DTT, and 1 μL M-MLV reverse transcriptase (ThermoFisher Scientific) were then added and cDNA conversion performed in a PCR thermocycler (37°C for 50 minutes, 70°C for 10 minutes). cDNA was amplified using RT-PCR with Brilliant III SYBR Green (Agilent) as per manufacturer's instructions. Ct analysis was performed using CFX Maestro software (BioRad). Reference genes RPL13A and GAPDH were chosen for experiments involving lymphoblast cells and SH-SY5Y cells respectively, for their relative stability between experiments in these cell lines (Hoerndli et al., 2004; Hruz et al., 2011). Relative mRNA expression values were then calculated using the $2^{-\Delta\Delta CT}$ method (Schmittgen and Livak, 2008). Low CAV2 expression leading to non-amplification in one cell line was assigned a maximum CT value of 40 for one repeat (McCall et al., 2014).

SH-SY5Y neuronal differentiation

Human SH-SY5Y neuroblastoma cells were seeded at densities of either $5x10^4$ cells per well of a 6-well culture plate, or $2x10^3$ cells per well of a 96-well culture plate in DMEM (Lonza) supplemented with 10% (v/v) FBS, 50 units/mL penicillin and 50 μg/mL of streptomycin. 24 hours after seeding the media was changed to DMEM supplemented with 5% (v/v) FBS, 50 units/mL penicillin, 50 μg/mL of streptomycin, 4mM l-glutamine and 10 μM retinoic acid. After 72 hours, the medium was switched to neurobasal media

(ThermoFisher Scientific) containing 1% (v/v) N-2 supplement 100x, 50 units/mL penicillin, 50 μg/mL of streptomycin, 1% l-glutamine and 50ng/mL human BDNF. Cells were cultured for an additional 3 days until fully differentiated.

To confirm neuronal differentiation blinded, semi-automated analysis of neurite length was performed using the SimpleNeurite-Tracer plugin for FIJI (Longair et al., 2011). 2D images were converted to 8-bit grayscale and successive points along the midline of a neural process were selected. The software automatically identified the path between the two points. Tracing accuracy was improved using Hessian-based analysis of image curvatures. The AnalyzeSkeleton plugin (Arganda-Carreras et al., 2010) was used to quantify the morphology of the traces.

Immunocytochemistry

SH-SY5Y cells were fixed with 4% paraformaldehyde for 15 minutes and washed 3x with PBS. Cells were blocked in 5% normal horse serum containing 0.1% Triton X-100 for 1 hour at RT. All primary antibodies were diluted in blocking solution (anti-Caveolin-1, 1:500; α-tubulin, 1:2000; anti-Pax6, 1:200). Cells were incubated in the primary antibody for 2 hours at RT and washed 3x in PBS before incubation in the appropriate secondary antibody (1:1000 in PBS) for 1 hour at RT. Nuclear counterstain (Hoechst 33342) was applied for 10 minutes followed by a 3x wash in PBS. Cells were imaged using an Opera Phenix High Content Screening System (PerkinElmer).

Live cell imaging

96-well culture plates were coated in plasma fibronectin (Merck) (5μg/mL in PBS) for 30 minutes prior to cell seeding. Excess fibronectin was removed immediately before seeding lymphoblastoid cells at a density of $2x10^4$ cells per well. Lymphoblastoid cells were cultured in supplemented RPMI media for 2 days prior to live-cell imaging. Media was removed and 5μg/mL labeling solution (Cholera Toxin Subunit B [CTxB] + Hanks Balanced Salt Solution [HBSS]) was added to cell-containing wells and incubated for 45 minutes at 37°C, 5% CO_2. Nuclear counterstain (Hoechst 33342) was applied for the final 5 minutes of the incubation. The labeling solution was removed, cells were washed 2x in PBS and incubated in 200μL pre-warmed HBSS for imaging. Live imaging was performed via confocal microscopy using an Opera Phenix™ High-Content Screening System (PerkinElmer) at 37°C, 5% CO_2. Cells were visualized at 40x magnification within a high-resolution z stack consisting of images at 0.5μm intervals through the entire nuclear volume of the cell.

QUANTIFICATION AND STATISTICAL ANALYSIS

Immunoblotting and quantitative PCR

Statistical analysis was conducted in GraphPad Prism 7 (La Jolla, CA). All bar graphs show the mean ± SD. To identify statistical differences between treatment groups utilized Welch's unpaired t test.

Burden testing

The optimal unified test (SKAT-O) was used to perform burden testing in enhancer regions because it is optimized for large numbers of samples and for regions where a significant number of variants may not be causal (Lee et al., 2012). SKAT tests upweight significance of rare variants according to a beta density function of MAF in which $w_j = Beta(p_j, a_1, a_2)$, where p_j is the estimated MAF for SNP_j using all cases and controls, and the parameters a_1 and a_2 are prespecified. Optimal values of a_1 and a_2 were chosen using TBK1 enhancers where it was hypothesized ALS-associated should be present. Increasing values of a_2 correspond to a relative upweighting of increasingly rare variants; optimum ALS-association was discovered for $a_2 = 250$ ($a_2 = 25$, p = 0.2; $a_2 = 250$, p = 0.003; $a_2 = 2500$, p = 0.01); therefore $a_2 = 250$ was chosen for all further statistical tests.

When variants are expected to have equivalent functional impact SKAT can lose power (Basu and Pan, 2011) and therefore in coding regions where variant filtering was more stringent and based on functional as well as population/evolution observations Firth logistic regression was preferred. To adjust for confounders including population structure, burden testing used the first ten eigenvectors generated by principal components analysis of common variant profiles, and sex as covariates.

For the secondary analysis utilizing 32,298 European non-Finnish controls rare-variant burden testing was applied as before, except that sex and eigenvectors were not available for use as covariates.

Modeling of TF binding

Candidate TF were identified from ChIP-sequencing data clusters via the UCSC genome browser (https://genome.ucsc.edu track: 'Transcription Factor ChIP-seq Clusters from ENCODE 3, version: 3 Nov 2018'). Clusters associated with chr7:116222625T > C were first identified, and then cross-referenced with other ALS-associated CAV enhancer variants (Table S3) and common TF identified. Changes in putative TF-binding capacity between wild-type, mutant and CRISPR/SpCas9-edited sequences associated with the chr7:116222625T > C mutation were then identified using position specific scoring matrices (http://rsat.sb-roscoff.fr/matrix-scan-quick_form.cgi ENCODE human TFs 2018 03: 'CEBPB_disc1', 'RAD21_disc1', 'CTCF_disc1'). Relative weights for each TF/sequence combination were compared.

Cell Reports, Volume *33*

Supplemental Information

Rare Variant Burden Analysis within Enhancers Identifies *CAV1* as an ALS Risk Gene

Johnathan Cooper-Knock, Sai Zhang, Kevin P. Kenna, Tobias Moll, John P. Franklin, Samantha Allen, Helia Ghahremani Nezhad, Alfredo Iacoangeli, Nancy Y. Yacovzada, Chen Eitan, Eran Hornstein, Eran Ehilak, Petra Celadova, Daniel Bose, Sali Farhan, Simon Fishilevich, Doron Lancet, Karen E. Morrison, Christopher E. Shaw, Ammar Al-Chalabi, Project MinE ALS Sequencing Consortium, Jan H. Veldink, Janine Kirby, Michael P. Snyder, and Pamela J. Shaw

Cell Reports, Volume *33*

Supplemental Information

Rare Variant Burden Analysis within Enhancers

Identifies *CAV1* as an ALS Risk Gene

Johnathan Cooper-Knock, Sai Zhang, Kevin P. Kenna, Tobias Moll, John P. Franklin, Samantha Allen, Helia Ghahremani Nezhad, Alfredo Iacoangeli, Nancy Y. Yacovzada, Chen Eitan, Eran Hornstein, Eran Ehilak, Petra Celadova, Daniel Bose, Sali Farhan, Simon Fishilevich, Doron Lancet, Karen E. Morrison, Christopher E. Shaw, Ammar Al-Chalabi, Project MinE ALS Sequencing Consortium, Jan H. Veldink, Janine Kirby, Michael P. Snyder, and Pamela J. Shaw

Table S1: Enhancer regions associated with CAV1/CAV2 or TBK1 expression in different tissues. Related to Figure 1

Gene Target	Chromosome	Start	Finish	Tissue
CAV1	chr7	116474804	116476617	Tissue-agnostic
CAV1	chr7	116057349	116058637	Tissue-agnostic
CAV1:CAV2	chr7	116202174	116202561	Tissue-agnostic
CAV1:CAV2	chr7	115898310	115902716	Tissue-agnostic
CAV1:CAV2	chr7	116215990	116219157	Tissue-agnostic
CAV1:CAV2	chr7	115992110	115999387	Tissue-agnostic
CAV1:CAV2	chr7	116185994	116188883	Tissue-agnostic
CAV1:CAV2	chr7	116209195	116214933	Tissue-agnostic
CAV1:CAV2	chr7	116196622	116201351	Tissue-agnostic
CAV1:CAV2	chr7	116149154	116154310	Tissue-agnostic
CAV1:CAV2	chr7	116220723	116224531	Tissue-agnostic
CAV1:CAV2	chr7	116033656	116036708	Tissue-agnostic
CAV2	chr7	116078147	116088487	Tissue-agnostic
CAV2	chr7	115954125	115959325	Tissue-agnostic
CAV2	chr7	116180927	116184847	Tissue-agnostic
CAV2	chr7	116231455	116233866	Tissue-agnostic
CAV2	chr7	116065132	116068292	Tissue-agnostic
CAV2	chr7	116094877	116096147	Tissue-agnostic
CAV2	chr7	116003024	116004444	Tissue-agnostic
CAV2	chr7	116071887	116076515	Tissue-agnostic
CAV2	chr7	116176051	116176914	Tissue-agnostic
CAV2	chr7	116261779	116262057	Tissue-agnostic
CAV2	chr7	116062851	116064462	Tissue-agnostic
CAV2	chr7	116134797	116136789	Tissue-agnostic
CAV2	chr7	116154567	116155823	Tissue-agnostic
CAV2	chr7	116104521	116107038	Tissue-agnostic
CAV1	chr7	115816693	115817206	CNS
CAV1	chr7	115853719	115854235	CNS
CAV1	chr7	115870180	115870885	CNS
CAV1	chr7	115871349	115873794	CNS
CAV1	chr7	115910552	115913260	CNS
CAV1	chr7	115913518	115914249	CNS
CAV1	chr7	115955578	115958367	CNS
CAV1	chr7	115967434	115969066	CNS
CAV1	chr7	115993415	115996411	CNS
CAV1	chr7	116034665	116036373	CNS
CAV1	chr7	116066587	116067242	CNS
CAV1	chr7	116073579	116076380	CNS
CAV1	chr7	116080916	116084930	CNS
CAV1	chr7	116094659	116096095	CNS
CAV1	chr7	116103858	116107331	CNS
CAV1	chr7	116118084	116119473	CNS
CAV1	chr7	116149961	116153401	CNS
CAV1	chr7	115898864	115900695	CNS

CAV1	chr7	115867068	115868903	CNS
CAV1	chr7	115996955	115998734	CNS
CAV1	chr7	116170083	116171009	CNS
CAV1	chr7	116175367	116176059	CNS
CAV1	chr7	116181441	116182810	CNS
CAV1	chr7	116182954	116183855	CNS
CAV1	chr7	116186806	116188332	CNS
CAV1	chr7	116195961	116196301	CNS
CAV1	chr7	116199565	116200132	CNS
CAV1	chr7	116206178	116207412	CNS
CAV1	chr7	116213507	116219235	CNS
CAV1	chr7	116221274	116225513	CNS
CAV1	chr7	116207998	116213360	CNS
CAV1	chr7	116227556	116229293	CNS
CAV1	chr7	116268838	116269440	CNS
CAV1	chr7	116273324	116274010	CNS
CAV1	chr7	116327276	116331188	CNS
CAV1	chr7	116331287	116334384	CNS
CAV1	chr7	116345275	116345781	CNS
CAV1	chr7	116345958	116349052	CNS
CAV1	chr7	116353124	116354955	CNS
CAV1	chr7	116355221	116357861	CNS
CAV1	chr7	116411044	116413659	CNS
CAV1	chr7	116415730	116419494	CNS
CAV1	chr7	116421735	116423410	CNS
CAV1	chr7	116341997	116343027	CNS
CAV1	chr7	116439854	116443746	CNS
CAV1	chr7	114429291	114431269	CNS
CAV1	chr7	114525449	114526553	CNS
CAV1	chr7	114529275	114530372	CNS
CAV1	chr7	114567780	114569045	CNS
CAV1	chr7	114569345	114571951	CNS
CAV1	chr7	114573849	114576684	CNS
CAV1	chr7	114583410	114585966	CNS
CAV1	chr7	114627975	114630075	CNS
CAV1	chr7	114648866	114650794	CNS
CAV1	chr7	114680511	114680894	CNS
CAV1	chr7	114870292	114872289	CNS
CAV1	chr7	114939946	114941012	CNS
CAV1	chr7	114992058	114993494	CNS
CAV1	chr7	115186013	115186673	CNS
CAV1	chr7	115301123	115304100	CNS
CAV1	chr7	115304366	115304820	CNS
CAV1	chr7	115310813	115313335	CNS
CAV1	chr7	115317812	115318384	CNS
CAV1	chr7	115318462	115319029	CNS
CAV1	chr7	115737105	115737568	CNS
CAV1	chr7	116502110	116504190	CNS

CAV1	chr7	116511658	116512938	CNS
CAV1	chr7	116551519	116552444	CNS
CAV1	chr7	116638294	116639676	CNS
CAV1	chr7	116645209	116646095	CNS
CAV1	chr7	116701473	116702466	CNS
CAV1	chr7	116738137	116738529	CNS
CAV1	chr7	116738883	116739229	CNS
CAV1	chr7	116764255	116764795	CNS
CAV1	chr7	116764919	116766070	CNS
CAV1	chr7	116771574	116774341	CNS
CAV1	chr7	116797868	116798377	CNS
CAV1	chr7	116869939	116871410	CNS
CAV1	chr7	116908705	116911053	CNS
CAV1	chr7	116923096	116924277	CNS
CAV1	chr7	117222645	117223108	CNS
CAV1	chr7	117305812	117306347	CNS
CAV1	chr7	117468242	117468792	CNS
CAV1	chr7	114458233	114460475	CNS
CAV1	chr7	114572709	114573331	CNS
CAV1	chr7	114785263	114786500	CNS
CAV1	chr7	115670883	115671400	CNS
CAV1	chr7	116899318	116900524	CNS
TBK1	chr12	64976982	64978649	Tissue-agnostic
TBK1	chr12	64849299	64852075	Tissue-agnostic
TBK1	chr12	64855048	64856317	Tissue-agnostic
TBK1	chr12	65194398	65194833	Tissue-agnostic
TBK1	chr12	65139887	65142519	Tissue-agnostic
TBK1	chr12	64349522	64350375	Tissue-agnostic
TBK1	chr12	64988381	64990575	Tissue-agnostic
TBK1	chr12	64852385	64854077	Tissue-agnostic
TBK1	chr12	65058182	65079989	Tissue-agnostic
TBK1	chr12	64479266	64484481	Tissue-agnostic
TBK1	chr12	64943421	64943781	Tissue-agnostic
TBK1	chr12	64953898	64955793	Tissue-agnostic
TBK1	chr12	64490881	64493995	Tissue-agnostic

Figure S1: qPCR measurement of TBK1 mRNA expression relative to mean expression in normal controls. Expression normalised relative to loading control. Related to Figure 1.

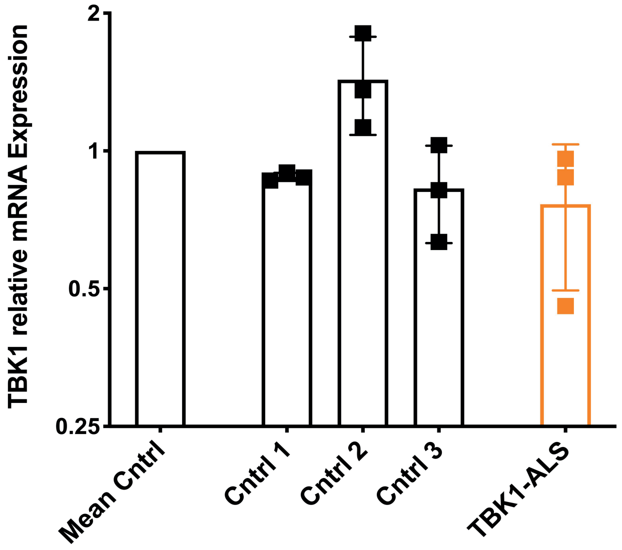

Table S2: Genetic variants wihin CAV1/CAV2 enhancer regions. D(i)=Discovery cohort; tissue-agnostic enhancers; D(ii)=Discovery cohort, CNS enhancers; R=Replicartion cohort.
Related to Figure 1.

Variants	ALS Frequency	Control Frequency	Cohort	Variants	ALS Frequency	Control Frequency	Cohort
chr7:115899988:T:C	0.00022	0	D(i)	chr7:115994719:G:A	0.000889878	0.001038961	D (ii)
chr7:115900605:A:G	0.00022	0	D(i)	chr7:115994770:C:G	0.000222469	0	D (ii)
chr7:115994269:C:T	0.00044	0	D(i)	chr7:115994869:C:T	0.000222469	0	D (ii)
chr7:115994303:G:A	0.00044	0	D(i)	chr7:115994875:C:T	0.000222469	0	D (ii)
chr7:115994310:A:G	0	0.00052	D(i)	chr7:115995110:A:G	0.000444939	0	D (ii)
chr7:115994595:T:G	0.00022	0	D(i)	chr7:115995386:G:A	0	0.000519481	D (ii)
chr7:115994627:T:C	0.00022	0	D(i)	chr7:115995411:C:G	0.000222469	0	D (ii)
chr7:115994719:G:A	0.00089	0.00104	D(i)	chr7:115996247:A:G	0.000222469	0	D (ii)
chr7:115994770:C:G	0.00022	0	D(i)	chr7:115996338:G:C	0.000222469	0	D (ii)
chr7:115994869:C:T	0.00022	0	D(i)	chr7:116035733:C:A	0.000444939	0	D (ii)
chr7:115994875:C:T	0.00022	0	D(i)	chr7:116152216:C:T	0.000222469	0.001038961	D (ii)
chr7:115995110:A:G	0.00044	0	D(i)	chr7:116175470:A:G	0.001557286	0.003116883	D (ii)
chr7:115995386:G:A	0	0.00052	D(i)	chr7:116175631:T:A	0	0.000519481	D (ii)
chr7:115995411:C:G	0.00022	0	D(i)	chr7:116175647:T:G	0.000222469	0	D (ii)
chr7:115996247:A:G	0.00022	0	D(i)	chr7:116181928:T:C	0	0.000519481	D (ii)
chr7:115996338:G:C	0.00022	0	D(i)	chr7:116182530:A:G	0.000222469	0	D (ii)
chr7:115996639:T:G	0.00022	0	D(i)	chr7:116182646:A:G	0.000667408	0.003116883	D (ii)
chr7:116034151:T:C	0.00022	0	D(i)	chr7:116182790:T:C	0	0.000519481	D (ii)
chr7:116035733:C:A	0.00044	0	D(i)	chr7:116211360:G:A	0.000444939	0.004675325	D (ii)
chr7:116152216:C:T	0.00022	0.00104	D(i)	chr7:116211574:C:T	0.000667408	0.000519481	D (ii)
chr7:116198357:C:T	0.00089	0	D(i)	chr7:116212628:A:G	0.000222469	0	D (ii)
chr7:116199522:T:C	0.00022	0	D(i)	chr7:116212861:A:G	0.000222469	0	D (ii)
chr7:116200589:T:A	0.00022	0	D(i)	chr7:116213876:A:T	0.000222469	0.001038961	D (ii)
chr7:116200705:C:T	0.00022	0	D(i)	chr7:116217283:C:G	0.000222469	0	D (ii)
chr7:116200719:C:A	0.00022	0	D(i)	chr7:116222625:T:C	0.000444939	0	D (ii)
chr7:116200953:A:T	0.00022	0	D(i)	chr7:116223328:A:G	0.000667408	0.000519481	D (ii)
chr7:116200959:G:T	0	0.00052	D(i)	chr7:116223448:C:T	0.000222469	0	D (ii)
chr7:116201160:T:C	0	0.00052	D(i)	chr7:116224152:G:A	0.002002225	0.007272727	D (ii)
chr7:116201160:T:G	0	0.00052	D(i)	chr7:116273953:C:T	0	0.000519481	D (ii)
chr7:116211360:G:A	0.00044	0.00468	D(i)	chr7:116273989:A:G	0.000222469	0.000519481	D (ii)
chr7:116211574:C:T	0.00067	0.00052	D(i)	chr7:116327730:T:C	0.000444939	0.000519481	D (ii)
chr7:116212628:A:G	0.00022	0	D(i)	chr7:116330812:T:C	0.000222469	0	D (ii)
chr7:116212861:A:G	0.00022	0	D(i)	chr7:116331129:T:C	0	0.000519481	D (ii)
chr7:116213876:A:T	0.00022	0.00104	D(i)	chr7:116333491:C:T	0.000222469	0	D (ii)
chr7:116217283:C:G	0.00022	0	D(i)	chr7:116333773:C:A	0	0.000519481	D (ii)
chr7:116222625:T:C	0.00044	0	D(i)	chr7:116342514:T:C	0.000222469	0	D (ii)
chr7:116223328:A:G	0.00067	0.00052	D(i)	chr7:116345315:A:G	0.000222469	0	D (ii)
chr7:116223448:C:T	0.00022	0	D(i)	chr7:116346600:T:C	0.000222469	0.000519481	D (ii)
chr7:116224152:G:A	0.00200	0.00727	D(i)	chr7:116347870:C:G	0.000222469	0	D (ii)
chr7:116474819:G:T	0.00067	0	D(i)	chr7:116355771:G:A	0.000222469	0	D (ii)
chr7:115957412:G:A	0.00022	0	D(i)	chr7:116356905:C:A	0.002892102	0.001038961	D (ii)
chr7:115957415:T:C	0.00022	0	D(i)	chr7:116356905:C:T	0.000222469	0.000519481	D (ii)
chr7:115957440:G:A	0.00022	0	D(i)	chr7:116357813:A:G	0.000444939	0	D (ii)

chr7:115957498:C:A	0.00022	0	D(i)	chr7:116412712:C:T	0.000222469	0	D (ii)
chr7:115957577:G:A	0.00022	0.00052	D(i)	chr7:116415873:G:T	0.000222469	0	D (ii)
chr7:116004321:G:A	0.00044	0	D(i)	chr7:116415881:T:G	0.000222469	0	D (ii)
chr7:116063605:A:G	0	0.00052	D(i)	chr7:116415938:C:G	0.000222469	0	D (ii)
chr7:116063624:T:C	0.00022	0	D(i)	chr7:116416799:C:T	0.001112347	0	D (ii)
chr7:116064199:T:C	0.00022	0	D(i)	chr7:116416941:A:G	0.000222469	0	D (ii)
chr7:116087637:G:A	0.00022	0	D(i)	chr7:116417019:A:G	0.000222469	0	D (ii)
chr7:116181928:T:C	0	0.00052	D(i)	chr7:116419236:G:T	0.001112347	0.001038961	D (ii)
chr7:116182530:A:G	0.00022	0	D(i)	chr7:116422352:C:T	0.000222469	0	D (ii)
chr7:116182646:A:G	0.00067	0.00312	D(i)	chr7:116422362:G:T	0.000222469	0	D (ii)
chr7:116182790:T:C	0	0.00052	D(i)	chr7:116422617:T:C	0.000889878	0.001558442	D (ii)
chr7:116184017:A:T	0.00912	0.00987	D(i)	chr7:116422623:C:A	0.005116796	0.002597403	D (ii)
chr7:116184130:T:A	0.00044	0	D(i)	chr7:116441514:T:C	0.000222469	0	D (ii)
chr7:116232009:G:C	0	0.00052	D(i)	chr7:116441539:C:T	0	0.000519481	D (ii)
chr7:116233640:T:C	0	0.00052	D(i)	chr7:116441559:C:A	0	0.000519481	D (ii)
chr7:114430787:C:G	0.008008899	0.008311688	D (ii)	chr7:116441604:C:A	0.003114572	0.001038961	D (ii)
chr7:114530323:G:A	0.000222469	0.000519481	D (ii)	chr7:116442636:T:A	0.000222469	0	D (ii)
chr7:114570572:C:A	0	0.000519481	D (ii)	chr7:116442680:C:T	0.000222469	0	D (ii)
chr7:114571016:G:A	0	0.000519481	D (ii)	chr7:116443482:C:T	0.000222469	0	D (ii)
chr7:114571375:G:C	0.000222469	0	D (ii)	chr7:116502532:G:C	0	0.001038961	D (ii)
chr7:114571427:G:T	0	0.000519481	D (ii)	chr7:116502617:C:G	0.000222469	0	D (ii)
chr7:114574678:G:A	0.000222469	0	D (ii)	chr7:116502985:G:C	0.000222469	0	D (ii)
chr7:114575111:A:C	0.001334816	0	D (ii)	chr7:116774049:T:C	0.000222469	0	D (ii)
chr7:114575748:A:G	0.000444939	0	D (ii)	chr7:116774169:C:T	0.000222469	0	D (ii)
chr7:114583529:T:C	0.000222469	0	D (ii)	chr7:116774262:G:T	0.004004449	0.000519481	D (ii)
chr7:114649995:C:A	0.000222469	0	D (ii)	chr7:116797872:G:A	0.000222469	0	D (ii)
chr7:114870400:T:G	0.000222469	0	D (ii)	chr7:116909821:G:A	0.000222469	0	D (ii)
chr7:114870987:C:T	0.000222469	0	D (ii)	chr7:116910136:C:A	0	0.000519481	D (ii)
chr7:114871000:G:C	0.000222469	0	D (ii)	chr7:116910162:C:G	0.000222469	0	D (ii)
chr7:114871004:A:T	0	0.000519481	D (ii)	chr7:116910505:A:C	0.000222469	0	D (ii)
chr7:114871090:C:T	0.000889878	0.001038961	D (ii)	chr7:117306108:T:C	0.000222469	0	D (ii)
chr7:114871798:T:C	0.000667408	0	D (ii)	chr7:117468457:C:T	0.000889878	0.001038961	D (ii)
chr7:114940467:A:G	0.000444939	0	D (ii)	chr7:117468773:A:G	0.000444939	0.000519481	D (ii)
chr7:114940537:T:C	0	0.000519481	D (ii)	chr7:114430749:G:T	0.000593472	0	R
chr7:114940606:C:T	0.000222469	0	D (ii)	chr7:114870400:T:G	0.001186944	0	R
chr7:115302267:G:A	0.000222469	0	D (ii)	chr7:114871090:C:T	0.000593472	0	R
chr7:115302388:C:T	0.000222469	0	D (ii)	chr7:114871798:T:C	0.000593472	0	R
chr7:115302422:A:G	0.000222469	0	D (ii)	chr7:114940077:A:G	0.000593472	0	R
chr7:115302425:G:T	0.000222469	0	D (ii)	chr7:114940422:T:A	0.000593472	0	R
chr7:115312834:C:T	0.000222469	0	D (ii)	chr7:115302422:A:G	0.000593472	0	R
chr7:115318030:G:A	0.000222469	0	D (ii)	chr7:115303637:A:G	0.000593472	0	R
chr7:115318066:G:A	0.001334816	0.000519481	D (ii)	chr7:115313234:T:G	0.000593472	0	R
chr7:115318198:G:A	0.000222469	0	D (ii)	chr7:115318066:G:A	0.002967359	0.002247191	R
chr7:115318232:C:T	0.000444939	0.000519481	D (ii)	chr7:115318495:C:T	0.000593472	0	R
chr7:115318257:A:T	0.000222469	0	D (ii)	chr7:115912442:G:A	0.000593472	0	R
chr7:115318267:C:G	0.000222469	0	D (ii)	chr7:115968420:C:T	0.001186944	0	R
chr7:115318323:G:C	0.000222469	0	D (ii)	chr7:115968431:A:G	0.000593472	0	R
chr7:115318339:G:A	0.000222469	0	D (ii)	chr7:115994330:C:T	0.000593472	0	R

chr7:115670928:A:G	0.001334816	0.001038961	D (ii)	chr7:115995441:A:C	0.000593472	0	R
chr7:115867898:G:A	0.000222469	0	D (ii)	chr7:116152067:C:T	0.000593472	0	R
chr7:115899988:T:C	0.000222469	0	D (ii)	chr7:116175470:A:G	0.000593472	0	R
chr7:115900605:A:G	0.000222469	0	D (ii)	chr7:116175798:A:G	0.000593472	0	R
chr7:115911421:A:G	0.000222469	0	D (ii)	chr7:116223751:C:G	0.001186944	0	R
chr7:115911791:T:G	0	0.000519481	D (ii)	chr7:116273806:G:A	0.000593472	0	R
chr7:115957412:G:A	0.000222469	0	D (ii)	chr7:116327730:T:C	0.000593472	0	R
chr7:115957415:T:C	0.000222469	0	D (ii)	chr7:116331111:T:C	0.000593472	0	R
chr7:115957440:G:A	0.000222469	0	D (ii)	chr7:116342272:T:C	0.000593472	0	R
chr7:115957498:C:A	0.000222469	0	D (ii)	chr7:116347329:T:C	0.000593472	0	R
chr7:115957577:G:A	0.000222469	0.000519481	D (ii)	chr7:116416799:C:T	0.000593472	0	R
chr7:115968420:C:T	0.000222469	0.000519481	D (ii)	chr7:116419236:G:T	0.001186944	0	R
chr7:115968429:G:A	0	0.000519481	D (ii)	chr7:116422617:T:C	0.000593472	0	R
chr7:115968452:C:T	0.000222469	0	D (ii)	chr7:116422623:C:A	0.007121662	0	R
chr7:115994269:C:T	0.000444939	0	D (ii)	chr7:116645747:A:C	0.000593472	0	R
chr7:115994303:G:A	0.000444939	0	D (ii)	chr7:116774262:G:T	0.005341246	0.004494382	R
chr7:115994310:A:G	0	0.000519481	D (ii)	chr7:116910136:C:A	0.000593472	0	R
chr7:115994595:T:G	0.000222469	0	D (ii)	chr7:117468457:C:T	0.000593472	0	R
chr7:115994627:T:C	0.000222469	0	D (ii)				

Figure S2: Increased dendrite length and altered PAX6 expression confirms successful neuronal differentiation of SH-SY5Y cells. Neuronal differentiated SH-SY5Y cells have increased dendrite length (A, *p<0.05, paired t-test, Forster et al., 2016). PAX6 is a transcription factor with an important role in neuronal development; reduced expression of PAX6 is concurrent with neuronal differentiation of Sh-SY5Y cells (B, Forster et al., 2016). Related to Figure 3**.**

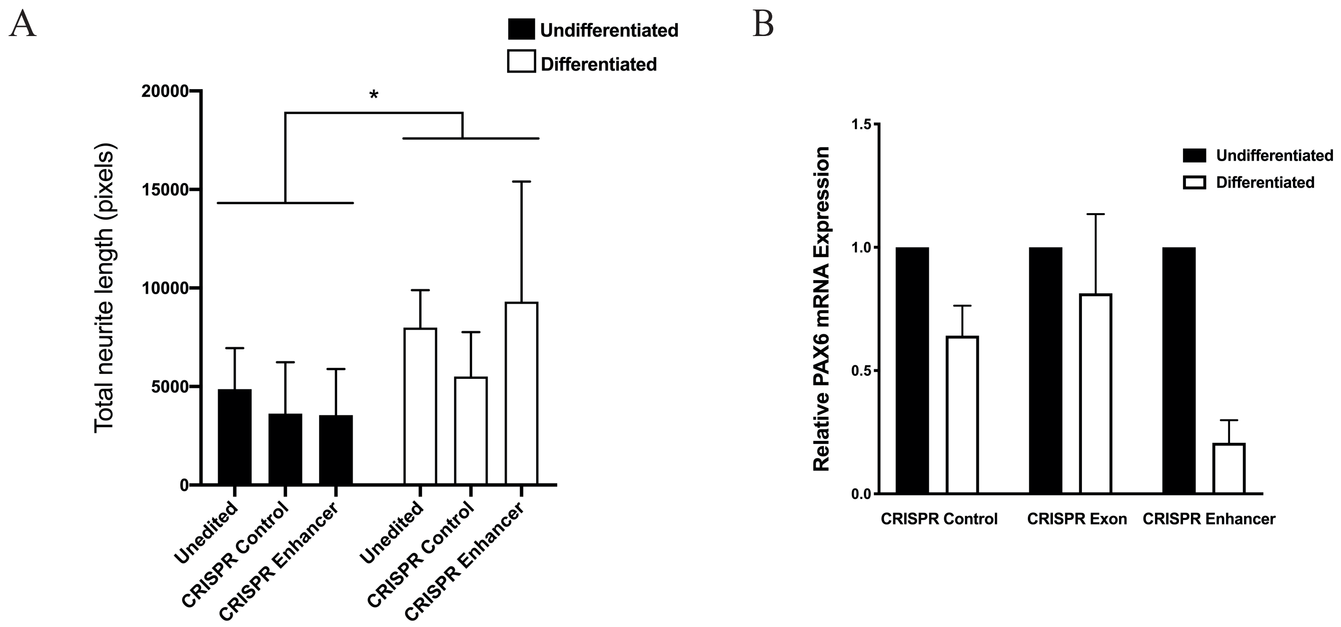

Table S3: DNA and RNA oligonucleotide sequences. Related to STAR Methods, 'CRISPR editing of mammalian cell lines'

**DNA oligos**	
qPCR RPL13A FWD	5'-CAAGCGGATGAACACCAACC-3'
qPCR RPL13A REV	5'-TTTTGTGGGGCAGCATACCT-3'
qPCR GAPDH FWD	5'-CAACTTTGGTATCGTGGAAGGAC-3'
qPCR GAPDH REV	5'-ACAGTCTTCTGGATGGCAGTG-3'
qPCR Pax6 FWD	5'-GTGCCCGTCCATCTTTGCTT-3'
qPCR Pax6 REV	5'-GCGCCCATCTGTTGGTTTTC-3'
qPCR CAV1 FWD	5’-CCAAGGAGATCGACCTGGTCAA– 3’
qPCR CAV1 REV	5’-GCCGTCAAAACTGTGTGTCCCT– 3’
qPCR CAV2	5'-TTCTCTTTGCCACCCTCAGCTG-3'
qPCR CAV2	5'-GAAGCATCGTCCTACGCTCGTA-3'
sequencing primers CAV enhancer 1 FWD	5'-ACCCTCCAGCACTAATGGACTT-3'
sequencing primers CAV enhancer 1 REV	5'-CCTGAGTTGATGACCCTTCTCCT-3'
sequencing primers CAV enhancer 2 FWD	5'-CTGCATACGCTATACCCGGC-3'
sequencing primers CAV enhancer 2 REV	5'-AGGTGTTTCGCTCCTCTGTC-3'
sequencing primers CAV exon FWD	5'-AGTACAGAGGGGTGTGGTGT-3'
sequencing primers CAV exon REV	5'-GGCTTACCTTGACCACGTCA-3'
**RNA Oligos (crRNA)**	
crRNA targeting CAV enhancer	5'-UUGUAAUCAGGAAUUUUCCA+modified linker-3'
crRNA targeting CAV exon	5'-AGUGUACGACGCGCACACCA+modified linker-3'

Table S4: Demographic information for patient-derived lymphoblastoid cell lines. Related to STAR Methods, 'Patient-derived lymphoblastoid cells'

	Genetic Variant	Age at collection (years)	Sex (M/F)
**CAV1-coding mutations**	p.Met111Val-*CAV1*	51	M
	p.Pro132Ala-*CAV1*	65	M
	p.Val155Ile-*CAV1*	50	F
**CAV1/CAV2-enhancer mutations**	chr7:116222625T>C	72	M
	chr7:115994269C>T	53	F
**TBK1-enhancer mutation**	chr12:65059913G>A	73	M
**ALS-controls**	-	78	M
	-	69	M
	-	49	F
**Neurologically normal controls**	-	48	F
	-	68	M
	-	56	M
